# Enhanced multistress tolerance of *Saccharomyces cerevisiae* with the sugar transporter-like protein Stl1^F427L^ mutation in the presence of glycerol

**DOI:** 10.1128/spectrum.00089-24

**Published:** 2024-12-16

**Authors:** Zixiong Liu, Lingling Shangguan, Linglong Xu, Huiyan Zhang, Wenxin Wang, Qiao Yang, Xiaoling Zhang, Lan Yao, Shihui Yang, Xiong Chen, Jun Dai

**Affiliations:** 1 Key Laboratory of Fermentation Engineering (Ministry of Education), Cooperative Innovation Center of Industrial Fermentation (Ministry of Education & Hubei Province), School of Bioengineering and Food Science, Hubei University of Technology, Wuhan, Hubei, China; 2 ABI Group, Donghai Laboratory, College of Marine Science and Technology, Zhejiang Ocean University, Zhoushan, Zhejiang, China; 3 State Key Laboratory of Biocatalysis and Enzyme Engineering, School of Life Sciences, Hubei University, Wuhan, Hubei, China; University of Mississippi, University, Mississippi, USA

**Keywords:** multistress tolerance, sugar transporter-like protein mutation, *Saccharomyces cerevisiae*, glycerol

## Abstract

During microbial industrial production, microorganisms often face diverse stressors, including organic solvents, high salinity, and high sugar levels. Enhancing microorganism tolerance to such stresses is crucial for producing high-value-added products. Previous studies on the mechanisms of 2-phenylethanol (2-PE) tolerance in *Saccharomyces cerevisiae* revealed a potential connection between the sugar transporter-like protein (Stl1) mutation (F427L) and increased tolerance to high sugar and salt stress, suggesting a broader role in multistress tolerance. Herein, we showed that the Stl1^F427L^ mutant strain (STL) exhibits significantly improved multistress tolerance in the presence of glycerol. Molecular dynamics simulations indicated that Stl1^F427L^ may enhance glycerol molecular binding, resulting in a significant increase in the intracellular glycerol content of the mutant strain STL. Additionally, under multistress conditions, pyruvate and ergosterol levels and catalase (CAT) and superoxide dismutase (SOD) activities were significantly increased in the mutant strain STL compared with the control strain 5D. This resulted in a notable increase in cell membrane toughness and a decrease in intracellular reactive oxygen species levels. These findings highlight the mechanism by which Stl1^F427L^ enhances *S. cerevisiae* tolerance to multistress. Importantly, they provide novel insights into and methodologies for improving the resilience of industrial microorganisms.

**IMPORTANCE:**

Stl1^F427L^ exhibits improved strain tolerance to multistress when adding glycerol, may enhance glycerol molecular binding, and can make a significant increase in intracellular glycerol content. It can reduce reactive oxygen species levels and increase ergosterol content. This paper provides novel insights and methods to get robust industrial microorganisms.

## INTRODUCTION

*Saccharomyces cerevisiae*, serving as the foundational microorganism in cell factories, is extensively applied in manufacturing high value-added “natural” products, such as biofuels and biochemical products (1–4). Although metabolically engineered microorganisms have been extensively used in industrial production, particularly in the synthesis and manufacturing of biobased chemicals, highlighting their potential and performance, their productivity has not fully met commercial needs. This shortfall presents a major obstacle to sustainable economic development (5, 6). Microbial industrial production is a highly complex and dynamic process, with numerous stress factors hindering microbial growth and metabolism (7). The inherent low tolerance of microorganisms to adverse stress factors is considered a primary reason for the challenge of achieving high productivity (8).

Notably, 2-phenylethanol (2-PE), a rose-scented aromatic alcohol, is the second most used spice worldwide and is widely used in food and cosmetics. Currently, microbial fermentation is used to produce 2-PE in line with the concept of “green and sustainable development.” In industrial 2-PE production processes involving *S. cerevisiae*, the organism inevitably encounters a range of adverse stress factors (9, 10), including high 2-PE concentrations (2, 11–13), high-glucose conditions (14), and high-salt (15) stress, all of which inhibit cell proliferation (16). Enhancing the tolerance of *S. cerevisiae* to such environmental stresses is critical for efficient bioproduction (17, 18). Therefore, investigating yeast stress tolerance mechanisms and strategies for developing robust yeast strains capable of withstanding multiple stressors is considered crucial.

Initially, we conducted an adaptive laboratory evolution analysis of *S. cerevisiae* CEN.PK113-7D to investigate 2-PE tolerance mechanisms. As a result, we identified over a dozen mutated genes potentially involved in tolerance to 2-PE stress, such as *PDR1^T2584C^, PKH1^2099delA^, PAU1^63delT^,* and *STL1^C1281G^*. However, among the mutant strains of the *PDR1*, *PKH1*, and *PAU1* genes, only the *PDR1* gene mutant strain PDR_862 has been verified to exhibit enhanced tolerance to 2-PE stress (2). The existing 2-PE tolerance genome sequencing data are akin to a vast ocean, teeming with genes that could potentially enhance the 2-PE tolerance of *S. cerevisiae* and even improve its broad-spectrum tolerance. Extensive research has shown that yeast strains initiate the high osmolarity glycerol (HOG) response pathway (19) when subjected to external stress, particularly hypertonic stress. This leads to the activation of glycerol synthetase genes (*GPP1* and *GPP2*) (20) for glycerol synthesis and glycerol transporter genes (*FPS1* and *STL1*) for glycerol export and import, respectively (21, 22). This allows the yeast cells to accumulate as much biocompatible solvent, glycerin, as possible to resist external stress. One of the key findings from previous studies is the role of the sugar transporter-like protein Stl1, also known as the glycerol/H^+^ transporter. Holyavkin et al. (17) found that 2-PE stress correlates with the HOG signaling pathway, which regulates Stl1 protein expression. Auesukaree et al. (23) and Jiménez-Martí et al. (24) found that the HOG pathway was activated in *S. cerevisiae* subjected to high sugar stress. Although these studies showed that the HOG response pathway is activated under high sugar stress, leading to the activation of its downstream glycerol/H^+^ transporter gene *STL1* (22)*,* there are few reports on the relationship between the activation of *STL1* and high sugar stress. There is a lack of research on the role of the Stl1 protein under high salt conditions in *S. cerevisiae*. Therefore, we sought to find similar functions of the Stl1 protein under the same adverse conditions in other yeasts, such as *Kluyveromyces marxianus*, *Debaryomyces hansenii*, and *Candida albicans*. Numerous studies have shown that when these yeasts are subjected to high salt conditions, Stl1 can transport extracellular glycerol into yeast cells, thereby enhancing their tolerance to extracellular stress (25–27). Although the function of glycerol transporters responsible for glycerol transportation has been extensively investigated, the relationship between functional changes resulting from certain amino acid mutations and stress tolerance remains unclear. Therefore, there is a need to examine the effect of the Stl1^F427L^ mutation on the tolerance of *S. cerevisiae* to 2-PE and high sugar or high salt stress as well as to further investigate the link between this mutation and altered tolerance to 2-PE, high sugar, and high salt stress.

Herein, we generated the mutant strain *S. cerevisiae* STL by transforming the Stl1 protein of the control strain *S. cerevisiae* CEN.PK113-5D into Stl1^F427L^. We investigated changes in 2-PE, high sugar, and high salt stress tolerance in *S. cerevisiae* after introducing the Stl1^F427L^ mutation, assessing the associations between these stressors and the mutant protein. Our findings revealed that *S. cerevisiae* exhibited significantly improved tolerance to 2-PE, high sugar, and high salt stress when glycerol was added. Bioinformatics and intracellular glycerol content analysis showed that in the presence of glycerol, the Stl1^F427L^ mutant protein displayed higher glycerol binding and transport capabilities compared with the wild-type Stl1. This resulted in increased glycerol uptake and a significant rise in intracellular glycerol levels. Changes in intracellular reactive oxygen species (ROS) levels and cell membrane components suggest that increased intracellular glycerol effectively alleviates oxidative stress through various pathways, including cytoprotective agents, reducing substances, and ergosterol anabolism. These findings highlight the relationship between structural and functional changes in the mutant transmembrane transport protein Stl1^F427L^ and stress tolerance, offering a novel concept and strategy for enhancing the robustness of *S. cerevisiae* in industrial production.

## MATERIALS AND METHODS

### Plasmids, strains, genes, materials, media, and growth conditions

*S. cerevisiae* CEN.PK113-5D (Mat*a* URA3-52 HIS3 LEU2 TRP1 MAL2-8c SUC2), hereafter referred to as the control strain 5D, was kept in a laboratory at Hubei University of Technology. The Stl1^F427L^ mutant strain (*S. cerevisiae* STL) was constructed using CRISPR/Cas9 gene-editing technology (28), hereafter referred to as the mutant strain STL targeting the *STL1* gene in the control strain 5D, converting a C base to a G base at position 1281. *Escherichia coli* JM110 (29) was used to construct gene-editing plasmids, and the plasmid pML104 (30) was employed for strain construction.

Key materials used were as follows: ampicillin (Guangzhou Saiguo Biotechnology Co., Ltd.); T_4_ DNA ligase and restriction enzymes *Smi*I and *Fba*I (Baori Medical Technology Co. Ltd., Beijing); 2×A_8_ polymerase chain reaction (PCR) master mix (Aidlai Biotechnology Co., Ltd., Beijing); Plasmid Fraction Extraction Kit, PCR Recovery and Cleaning Kit, Yeast Genome DNA Extraction Kit (Tiangen Biochemical Technology Co., Ltd.); Liquid Glycerin Kit (Pulilai Gene Technology Co., Ltd., Beijing); and 5-fluorouracetic acid (BBI Life Sciences Ltd.). All other reagents used were analytically pure.

The yeast strain was activated using yeast extract peptone dextrose (YPD) medium (20 g/L glucose, 20 g/L peptone, and 10 g/L yeast extract). Stress conditions were induced by adding 3.0 g/L 2-PE, 700 g/L glucose, and 60 g/L NaCl to the YPD medium. Plasmid enrichment was performed using lysogeny broth medium (5 g/L yeast extract, 10 g/L tryptophan, 10 g/L NaCl, and pH adjusted to 7.4 using NaOH), with corresponding antibiotics added as needed. Positive-transformed yeast strains were screened using SC medium without uracil (20 g/L glucose, 6.7 g/L yeast nitrogen base without amino acids, and a 2 g/L mixture containing trace amounts of various amino acids). Plasmid loss screening for pML104 was performed using SC medium with 1 g/L 5-fluoro-oriotic acid (20 g/L glucose, 6.7 g/L yeast nitrogen base without amino acids, a 2 g/L mixture containing trace amounts of various amino acids, 0.2 g/L uracil, 1 g/L 5-fluoro-oriotic acid, and 23 g/L agar).

### Mutant strain STL construction

The primers used for the *STL1^C1281G^* mutation are listed in Table S1. The steps for constructing the Stl1^F427L^ mutant STL were as follows. First, using DNA from the control strain 5D as a template, *STL1* U-F/*STL1* U-R and *STL1* D-F/*STL1* D-R primers were employed for PCR amplification to obtain upstream and downstream homologous fragments. Using the isomolar mixed upstream and downstream homologous fragment as a template along with *STL1* U-F and *STL1* D-R primers, the fusion fragment, namely donor DNA, was amplified via fusion PCR (Fig. S1). Second, the pML104 plasmid was linearized using *Fba*I and *Smi*I enzymes, followed by hybridization with oligochain fragments of *STL1* gRNA-F and *STL1* gRNA-R, generating gRNA to target *STL1* recognition. T_4_ ligase was used to attach the linearized pML104 plasmid to the hybridized gRNA, yielding the recombinant plasmid pML104-gRNA. Third, donor DNA and the recombinant plasmid pML104-gRNA were transformed into receptive cells of the control strain 5D via LiAc transformation, and positive clones were screened using uracil plates and subjected to sequencing (31) (Fig. S2 and S3). Positive colonies were screened for pML104 plasmid loss mutants using 5-fluoro-oriotic acid plates (32). Successful plasmid loss was verified by culturing in a liquid medium lacking uracil. A yeast strain with a beneficial mutation was randomly selected and designated as the mutant strain STL.

### Spot assay

The *S. cerevisiae* was cultured in the YPD medium until it reached the log phase of growth. The cells were then harvested using centrifugation at 12,000 r/min for 1 min and resuspended in sterile water. Following this, the cells were diluted to an OD_600 nm_ of 1 using sterile water. The yeast cells were then serially diluted 10-fold and spotted on various media: YPD, 2.5 g/L 2-PE–YPD, 3.0 g/L 2-PE–YPD, 500 g/L glucose–YPD, 700 g/L glucose–YPD, 40 g/L NaCl–YPD and 60 g/L NaCl–YPD. These media were supplemented with either 20 g/L glycerol or no glycerol. The cultures were then incubated at 30°C for 2–5 days.

Due to the reflection of the plate medium, there are factors unrelated to experimental results such as tiny lamp reflection and large area shadow in our original picture data (Fig. S12). In order to reduce these unrelated factors affecting the observation of experimental results, we utilized Adobe Photoshop CC software (version 2019.0.0, Adobe Systems, Inc.) to adjust the background color of the original picture in each stess condition to black to highlight the the colonies features in experimental results, based on the following procedure: (i) import the original picture data taken and circle the colonies part of the plate with the lasso tool; (ii) click “Select and Cover” in “Select” in the upper toolbar and adjust the excess background color to disappear by adjusting the edge brush tool; (iii) select “Clean Color” in the “Reset Select and Cover” property bar and output to “New Layer with Layer Mask”; (iv) create a new solid color layer, change its color to black, and put it under the “New Layer with Layer Mask” layer; (v) fine-tune the “New Layer with Layer Mask” by adjusting the exposure, displacement, brightness, and color level and watch the image change while adjusting the parameters until the final image has a pure black background and the colonies are clearly visible.

### Physiological parameters of strains under different stress conditions

The activated control strain 5D and mutant strain STL were inoculated into media comprising YPD, 3.0 g/L 2-PE–YPD, 700 g/L glucose–YPD, and 60 g/L NaCl–YPD. These media were supplemented with either 20 g/L glycerol or no glycerol. The strains were fermented at 30°C and 200 r/min, with samples collected every 6 h before 24 h of fermentation and then at 12 h intervals. These samples were centrifuged at 4°C and 5,000 r/min for 10 min, and both the supernatant and yeast cells were stored at −20°C for subsequent growth parameter analysis.

The yeast was cultured to a stable phase in YPD, 3.0 g/L 2-PE–YPD, 700 g/L glucose–YPD, and 60 g/L NaCl–YPD. These media were supplemented with either 20 g/L glycerol or no glycerol. The optical density of yeast cells at a 600 nm wavelength (OD_600 nm_) was determined using a spectrophotometer. Intracellular and extracellular glycerin concentrations (mg/L) were quantified using a glycerol kit (Pulitzer Gene Technology Co., Ltd., Beijing). Yeast cells were desiccated for 12 h, and the dried mass of these cells was determined. The yeast dry mass concentration (g DCW/L) was calculated as the ratio of cell dry mass to the culture volume containing yeast cells. Intracellular glycerol content (mg/g DCW) was calculated as the ratio of intracellular glycerol concentration to cell dry mass concentration, whereas extracellular glycerol content (mg/L) represented the extracellular glycerol concentration.

The yeast was incubated until it reached the stable phase in various media: YPD, 3.0 g/L 2-PE–YPD, 700 g/L glucose–YPD, and 60 g/L NaCl–YPD. These media were supplemented with either 20 g/L glycerol or no glycerol. Intracellular ROS levels were measured (33) using the intracellular accumulation of ROS to oxidize intracellular lipase, resulting in the formation of 2′,7′-dichlorodihydrofluorescein diacetate (DCFH-DA) to obtain 2′,7′-indichlorofluorescent diacetate, which emits green fluorescence (660 nm) when exposed to a laser. For this purpose, cells from the control strain 5D and mutant strain STL were cultured under various stress and glycerol conditions. These cells were diluted to OD_600 nm_ = 0.2, washed three times with phosphate-buffered saline, filtered through a 400-mesh screen, and stained with 5 µL of 0.02 mM DCFH-DA at 4°C for 30 min. A BD fluorescence-activated cell sorting flow cytometer (BD Biosciences, New Jersey, USA) was utilized to measure the fluorescence of the samples. For each sample, 10,000 cells were analyzed. All data were subsequently analyzed using FlowJo software (FlowJo-V10, USA).

The yeast was incubated until it reached the stable phase in various media: YPD, 3.0 g/L 2-PE–YPD, 700 g/L glucose–YPD, and 60 g/L NaCl–YPD. These media were supplemented with either 20 g/L glycerol or no glycerol. Following this, the total ergosterol content was determined as per the method described by de Oliveira et al. (21) by sampling 5 mL of fermentation liquid from cultures of the control strain 5D and mutant strain STL under different stress and glycerol conditions. After centrifugation at 12,000 r/min for 5 min, the supernatant was removed and the yeast cells were washed three times with deionized water. Subsequently, 10 mL of a 20% NaOH solution and 5 mL of 95% ethanol were added for saponification in a 90°C water bath for 1.5 h. An additional 2 mL of 95% ethanol was then added, and the saponification continued for 1 h. Thereafter, 12.5 mL of ethyl acetate was added and shaken vigorously for 1 min. The solution was allowed to stand for approximately 30 min until clear stratification occurred. The upper layer was extracted, and OD_282 nm_ was measured. The total ergosterol concentration (mg/L) was converted to OD_282 nm_ based on the ergosterol standard curve. Total ergosterol content (mg/g DCW) was calculated as the ratio of total ergosterol concentration to yeast dry mass concentration.

The yeast was cultured until it reached the stable phase in various media: YPD, 3.0 g/L 2-PE–YPD, 700 g/L glucose–YPD, and 60 g/L NaCl–YPD. These media were supplemented with either 20 g/L glycerol or no glycerol. Following this, fatty acid-related indices (34) were determined as follows. An appropriate amount of the sample was weighed in a 50 mL centrifuge tube, and 15 mL of a potassium hydroxide–methanol solution (10% potassium hydroxide and 5% sterile water) was added for saponification and reflux in an 80°C water bath for 2 h. After the reaction, the saponification solution was transferred to a clean 50 mL centrifuge tube and 5 mL of 6 mol/L hydrochloric acid solution was added for acidification. The tube was sealed and shaken thoroughly, followed by the addition of 10 mL of *n*-hexane. The mixture was placed in a shaker for extraction for 3 h at 185 r/min, and the upper organic phase was then transferred to a clean 50 mL centrifuge tube and dried using nitrogen for the subsequent methyl ester reaction. This involved adding 1 mL of 15% boron trifluoride methanol solution, transferring to an 80°C (constant temperature) water bath for 10 min, cooling at room temperature, adding 1 mL of 0.05 mol/L potassium hydroxide–methanol solution and reheating at 80°C (constant temperature) in a water bath for 10 min, and finally adding 5 mL of saturated sodium chloride aqueous solution after reaction completion. The sample was then extracted using 1 mL of *n*-hexane, and after removing the supernatant, an appropriate amount of anhydrous sodium sulfate was added. The sample was centrifuged, and the supernatant was removed. Gas chromatography was used for detection under the following conditions: column type: DB-225 (30 m × 0.25 mm × 0.25 µm); gasification chamber temperature: 250°C; detector temperature: 250°C; column temperature: 35°C; carrier gas: high-purity nitrogen (purity >99.99%); shunt ratio: 10:1; sample size: 1 µL; and constant linear velocity: 15.7 mL/min. The temperature procedure used in the cylindrical temperature chamber is provided in Table S2. Target fatty acid determination followed the guidelines of *GB 5009.168–2016 Determination of fatty acids in food of National Food Standard*. Total fatty acid content (μg/g DCW) was calculated as the ratio of the total fatty acid concentration to the yeast dry mass concentration, whereas unsaturated fatty acids/saturated fatty acids were determined as the ratio of the total unsaturated fatty acid content to the total saturated fatty acid content.

Determining intracellular glucose content during the logarithmic stage required collecting a 1 mL sample from the fermentation solution. After centrifugation at 12,000 r/min for 5 min, the precipitate was collected and subjected to cell disruption using an ultrasonic disruptor. The resulting cell lysate was centrifuged at 8,000 r/min at 4°C for 10 min, and the glucose concentration in the supernatant was measured using a biosensor (35). Yeast cells were dried for 12 h and weighed to determine the dry cell mass. The dry cell mass concentration (g DCW/L) was calculated as the dry cell mass (mg DCW) divided by the liquid cell volume (mL). Logarithmic intracellular glucose content (mg/g DCW) was computed as the intracellular glucose concentration (mg/L) divided by the dry cell mass concentration (g DCW/L).

Determining intracellular pyruvate content involved using a pyruvate detection kit (Biocaxis Biotechnology [Zhenjiang] Co., Ltd., Jiangsu). During the stable period, a 1 mL sample was collected from the fermentation solution, centrifuged at 12,000 r/min for 5 min, and the cells were washed three times with ddH_2_O. The extraction solution was mixed with yeast cells in appropriate proportions, subjected to ultrasound, incubated for 30 min, and then centrifuged at 8,000 r/min at 25°C for 10 min. The pyruvate concentration was measured in the supernatant. The yeast cells were dried for 12 h and weighed to determine the dry cell mass. The dry cell mass concentration (g DCW/L) was calculated as described earlier. Stable intracellular pyruvate content (mg/g DCW) was determined as the intracellular pyruvate concentration (mg/L) divided by the dry cell mass concentration (g DCW/L).

Catalase (CAT) activity was determined using a CAT detection kit (Guangzhou Saiguo Biotech Co., Ltd., Guangzhou). During the stable stage, a 1 mL sample was collected from the fermentation solution, centrifuged at 12,000 r/min for 5 min, mixed with the extract and yeast cells at an appropriate ratio, subjected to ultrasound for cell disruption, and then centrifuged at 8,000 r/min at 4°C for 10 min. The supernatant was used for enzyme activity determination.

Superoxide dismutase (SOD) activity was determined using a total superoxide dismutase test kit (Beijing Boxbio Science & Technology Co., Ltd., Beijing). At the stable stage, a 1 mL sample was collected from the fermentation solution and centrifuged at 1,000–2,000 r/min at 4°C for 10 min to collect cells. The cells were washed with precooled phosphate-buffered saline (PBS) or normal saline at 4°C once or twice and centrifuged at 8,000 r/min for 10 min, followed by collecting the precipitate. The cells were then disrupted via ultrasonication in precooled PBS at 4°C or in an ice bath. Finally, the homogenate was centrifuged at 12,000 r/min at 4°C for 5 min, and the supernatant was used for enzyme activity determination.

### Observations of cell morphology

To observe cell morphology, a 1 mL sample was collected from the fermentation solution during the logarithmic stage. Following centrifugation at 12,000 r/min for 5 min, the precipitate was collected. It was then suspended in a 2 mL EP tube with 1 mL of distilled water and centrifugally washed at 12,000 r/min for 3 min. A suitable amount of yeast cells was fixed in a 5% glutaraldehyde solution for 25 min, followed by centrifugation at 12,000 r/min for 3 min. The cells were washed twice with 0.01 mol/L PBS, allowed to stand for 10 min, and then centrifuged at 12,000 r/min for 2 min to remove the supernatant. Ethanol gradient dehydration was performed at 30%, 50%, 70%, 90%, and 100% ethanol for 10 min each, after which the supernatant was discarded. The dehydrated cells were placed in an oven at 70°C for 12 h and then sprayed with gold for scanning electron microscopy (SEM) observation. A SEM was used to examine the surface morphology of *S. cerevisiae* cells under multistress conditions (36).

### Real-time quantitative PCR

The control strain 5D and the mutant strain STL were inoculated in the YPD medium and cultured overnight on a shaking table at 200 r/min at 30°C. Exponential yeast cells were collected to prepare the inoculum and inoculated into the YPD medium containing 20 g/L glycerol before being cultured under the aforementioned conditions. Simultaneously, OD_600 nm_ was monitored to track cell density. When OD_600 nm_ reached approximately 0.5, the following were added to the medium: 3.0 g/L 2-PE, 700 g/L glucose, and 60 g/L NaCl. The cultures were grown to the later logarithmic stage as the experimental group, and cell samples under the corresponding conditions were obtained via centrifugation at room temperature and 4,000 r/min for 2 min.

When the mutant strain STL and control strain 5D reached the logarithmic stage, cell samples cultured in the YPD medium containing 20 g/L glycerol without stress agents were used as the control group, with centrifugation conditions identical to those of the experimental group. The cell precipitate was immediately frozen in liquid nitrogen and stored at −80°C until use. Prior to real-time quantitative PCR (RT-qPCR) analysis, total RNA extraction from cell samples was conducted according to the protocol described by Liu et al. (37). The extracted RNA was then purified using an RNA clean kit (Vazyme Biotech Co., Ltd., Nanjing), and the concentration was determined. Subsequently, reverse transcriptional reactions were performed according to Lewis’ procedure (38). For the RT-qPCR process, HiScript II QRT SuperMix and ChamQ Universal SYBR qPCR Master Mix were employed. The primers used for RT-qPCR are shown in Table S3. RT-qPCR data were analyzed using the model described by Liu et al. (39). Two biological experiments were repeated for each condition.

### Structural model

The amino acid sequence of the Stl1 protein was obtained from the National Center for Biotechnology Information (https://www.ncbi.nlm.nih.gov/protein) in FASTA format. In the mutant protein Stl1^F427L^, leucine (L) replaced phenylalanine (F) at residue 427 of the Stl1 protein’s amino acid sequence. The PDB file corresponding to the Stl1 protein was obtained from P39932·STL1_YEAST (AlphaFoldDB: AF-P39932-F1) in the UniProt database (https://www.uniprot.org/). The mutant Stl1^F427L^ PDB file with the three-dimensional structure of the Stl1 protein was obtained through DynaMut (40) (https://biosig.lab.uq.edu.au/dynamut/analysis).

Furthermore, the reliability of the models was established after importing them into the evaluation software SAVES v6.017 (https://saves.mbi.ucla.edu/) to evaluate the Ramachandran plot and validate molecular docking of the models. ChemDraw v20.0 (Cambridge Soft, USA) was used to illustrate the planar structure of the glycerol molecule, and Chem 3D was employed to construct its three-dimensional structure. The glycerol structure was optimized by calculating its minimum energy, resulting in the acquisition of the corresponding PDB file.

### Bioinformatics analysis

To determine the molecular weight, isoelectric point, negative and positive charge residues, instability index, hydropathicity index, and grand average of hydropathicity of the Stl1 protein and its mutant Stl1^F427L^ and to discern inherent attributes and subtle differences between them, ExPASy ProtParam tools (https://web.expasy.org/protparam/) were used for the online analysis of both the physical and chemical properties of the proteins (41).

For mastering Stl1 protein amino acids and its mutant Stl1^F427L^ each position the difference hydrophobicity, the Protscale website (http://web.expasy.org/protscale) was used to analyze hydrophobicity Stl1 and Stl1^F427L^ protein (42).

To predict the effect of the F-to-L mutation at residue 427 of the amino acid sequence of Stl1 protein on its 2D structure, we employed the SOPMA secondary structure prediction method (https://npsa-prabi.ibcp.fr/cgi-bin/npsa_automat.pl?page=/NPSA/npsa_sopma.html). The secondary structure distribution of all amino acid sequences of Stl1^F427L^, such as α-helix, extended chain, β-fold, and random curl, was determined using this method, and the results were compared with those of the Stl1 protein (43, 44).

To analyze the tertiary structures of the Stl1 and mutant Stl1^F427L^ and the differences between them, UCSF Chimera X was used to visualize their three-dimensional structure and structural alignment (45). I-TASSER (https://zhanglab.ccmb.med.umich.edu/I-TASSER/) was used to evaluate the accuracy of the Stl1 and Stl1^F427L^ topologies (46).

To assess the difference in surface charge distribution between Stl1 and Stl1^F427L^, the POLYVIEW-3D website (http://polyview.cchmc.org/polyview3d.html) was used for online prediction analysis. This website provides a space-filling structure of the two proteins (47).

To understand the effect of the change in a specific amino acid change(F427L) in Stl1 protein on the transmembrane structure domain, we ultilized TMHMM (http://www.cbs.dtu.dk/services/TMHMM/) to predict the differences in the transmembrane domains of the mutant and non-mutanted proteins. Finally, the structures of the two proteins were compared (48).

### Molecular dynamics simulation

UCSF Chimera X and Auto Dock-4 (44, 49) were used to predict the flexible docking of the Stl1 and Stl1^F427L^ proteins with glycerol molecules, respectively. The visualization of the docking results and analysis of the two-dimensional diagram of the protein–small molecule interactions in the complex-binding pocket of Stl1 and Stl1^F427L^ in relation to glycerol using UCSF Chimera X and Molecular Operating Environment (MOE) (45, 50). The molecular dynamics of the optimal complexes of Stl1 and Stl1^F427L^ proteins and glycerol obtained by molecular docking (51) were molecular dynamics simulated using the Gromacs2020 software. The Amber14sb force was selected as the protein force field and Gaff2 as the coordination force field; the SPC/E water model was used to add solvents to the protein–ligand system, and a water box with a periodic boundary of 1.2 nm was established (52). The particle mesh Ewald (PME) method was utilized to compute long-range electrostatic interactions. Furthermore, to neutralize the charge of the system, an appropriate number of sodium and chloride ions were introduced using the Monte Carlo ion placement method (52).

Prior to the formal simulation, energy minimization and equilibration was conducted using the following three steps: (i) the energy minimization of each system was performed using the steepest descent algorithm with 50,000 steps (minimization stopped when the maximum force was <1,000 kJ/mol). (ii) Each system underwent a 50,000-step NVT ensemble simulation while maintaining the number of particles, a temperature of 310 K, and a time step of 2 fs. (iii) A 50,000-step NPT ensemble simulation was conducted for each system at a temperature of 310 K, pressure of 1 atm, and a time step of 2 fs. The simulation was then continued for 200 ns, whereby the coordinates were saved every 10 ps for analysis (52).

The analysis included an examination of the root mean square deviation (RMSD), root mean square fluctuation (RMSF), radius of rotation (Rg), and changes in hydrogen bonds, solvent-accessible surface area (SASA), molecular mechanics–Poisson–Boltzmann surface area (MM-PBSA) binding energy, and energy contributions from amino acids involved in binding (residue–energy) between Stl1 and Stl1^F427L^ and glycerol molecules. We also conducted structural comparisons of the Stl1–glycerol and Stl1^F427L^–glycerol complexes at 0, 50, 100, 150, and 200 ns as the time points. Additionally, Gibbs free energy diagrams were generated based on molecular dynamics simulation trajectories.

### Data and statistics

The experiment to determine physiological parameters was replicated three times. Data are displayed as the arithmetic mean ± standard deviations. One-way analysis of variance (ANOVA) was employed to compare multiple groups with a control group, while one-way ANOVA with Tukey’s test was used for comparing multiple groups with a median group. A *P* value less than 0.05 was considered statistically significant. Origin 2021b software was used for data visualization.

## RESULTS

### Enhancement of stress tolerance through the Stl1^F427L^ mutation in the presence of glycerol

Previous experiments have established the potential of the Stl1^F427L^ mutant in a glycerol transporter (Stl1) that is closely associated with high sugar and salt tolerance to improve 2-PE tolerance (2). Based on the functional characteristics of Stl1^F427L^ and Stl1, we established groups with and without glycerol to investigate its effects on the growth of the control and mutant strains under 2-PE, high salt, and high sugar stresses.

**Fig 1:**
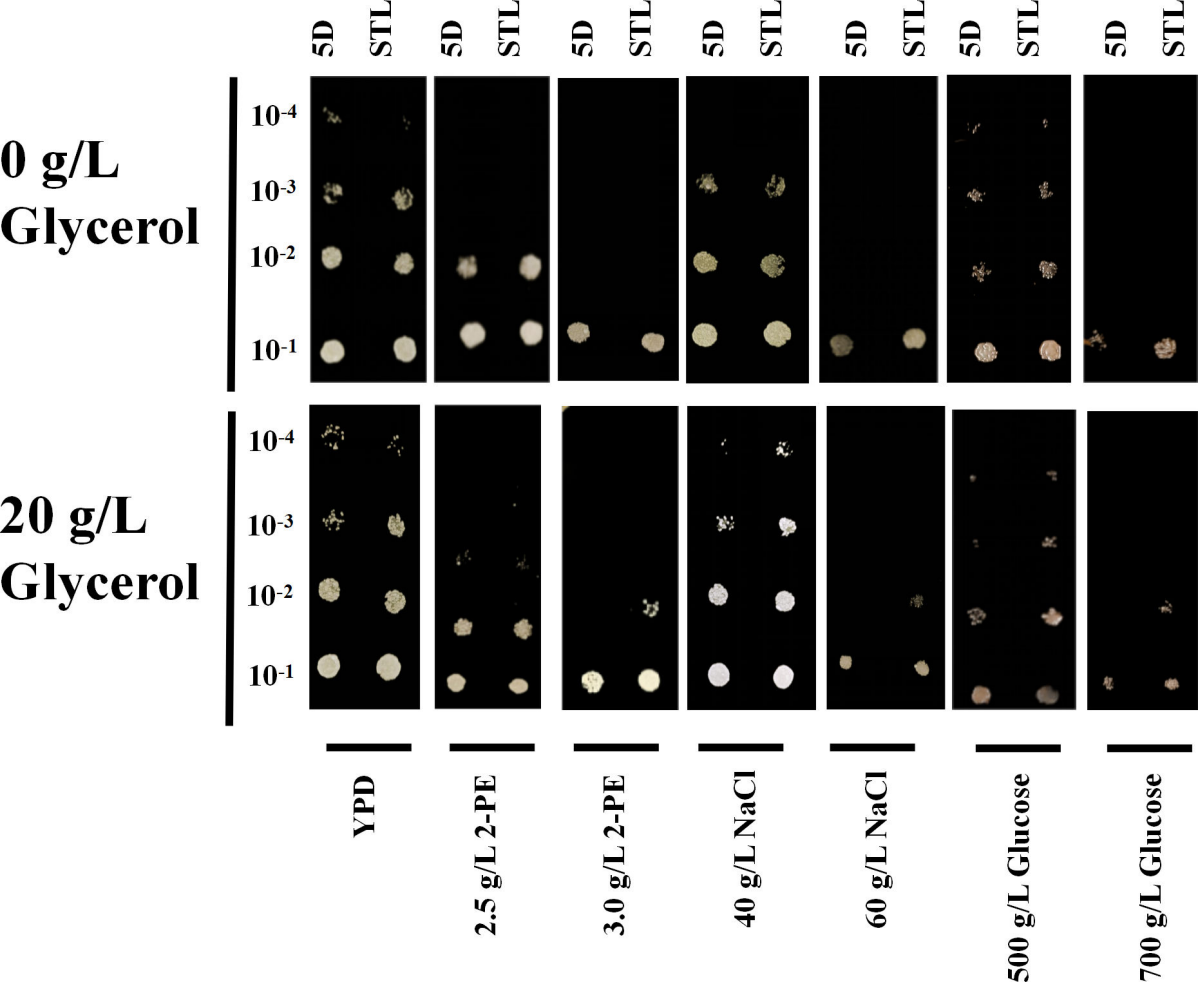
Identification of tolerance in the mutant strain STL. The unprocessed images are placed in Fig. S12 of supplemental figures.

**Fig 2:**
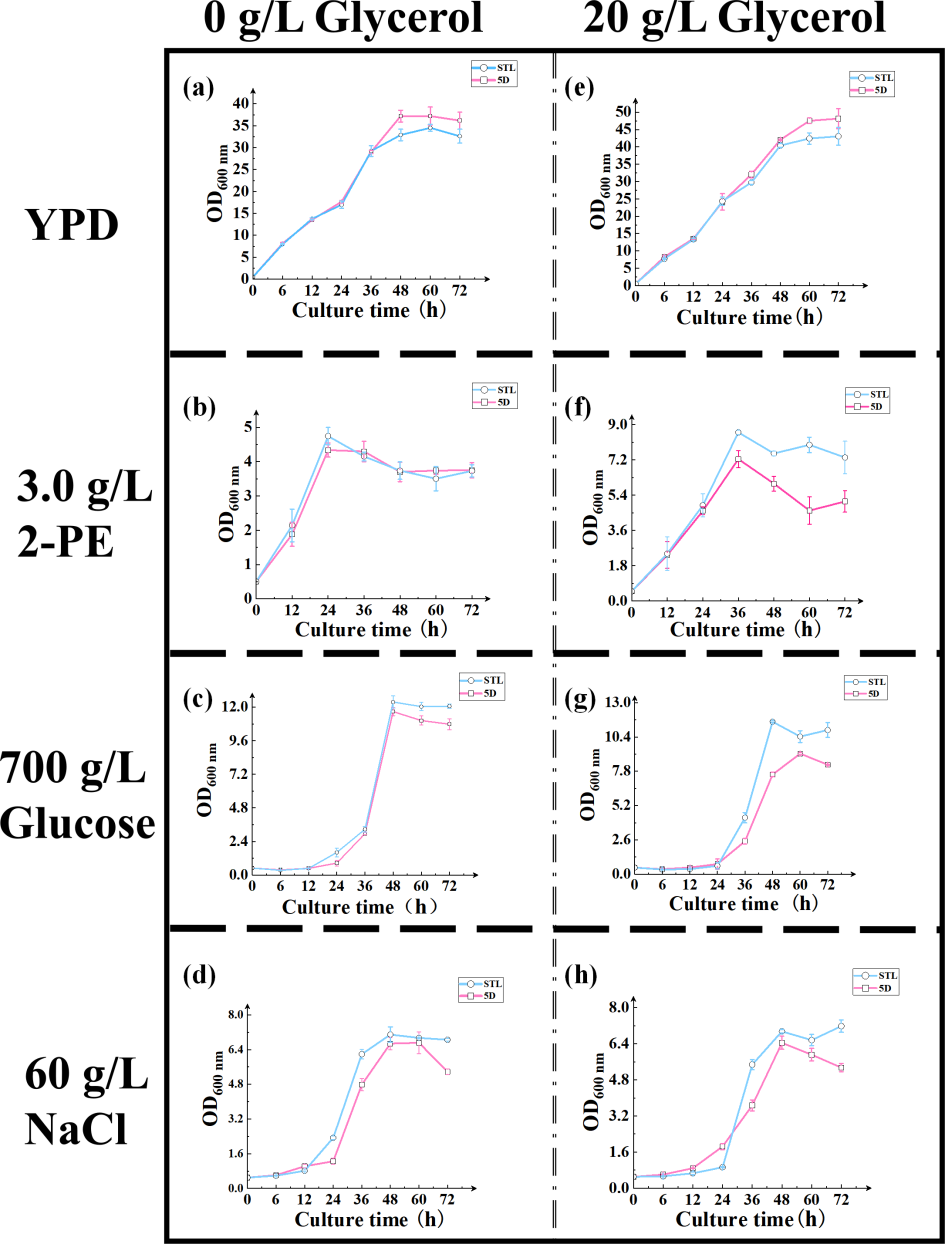
Comparison of the growth curve of the control strain 5D and mutant strain STL under various stress conditions. (**a and e**) Growth curve of (**a**) 0 g/L glycerol and (**e**) 20 g/L glycerol in YPD media cultures of the control and mutant strains; (**b and f**) growth curve of (**b**) 0 g/L glycerol and (**f**) 20 g/L glycerol in YPD media cultures with 3.0 g/L 2-PE for the control and mutant strains; (**c and g**) growth curve of (**c**) 0 g/L glycerol and (**g**) 20 g/L glycerol in YPD media cultures with 700 g/L glucose for the control and mutant strains; (**d and h**) growth curve of (**d**) 0 g/L glycerol and (**h**) 20 g/L glycerol in YPD media cultures with 60 g/L NaCl for the control and mutant strains. In all cases, OD_600 nm_ was quantified using an ultraviolet-visible spectrophotometer.

The plate assay and growth curve results revealed that the mutant strain STL exhibited superior colony growth when supplemented with 20 g/L glycerol compared with the control strain 5D under 3.0 g/L 2-PE, 60 g/L NaCl, and 700 g/L glucose stresses (Fig. 1 and 2). This enhancement was reflected in the maximum OD_600 nm_ (and the specific growth rate) values of 8.60 ± 0.31 (and 0.079 ± 0.0010 h^−1^), 7.09 ± 0.26 (and 0.084 ± 0.0005 h^−1^), and 11.56 ± 0.07 (and 0.093 ± 0.0003 h^−1^), which represented 19% (and 7%), 13% (and 53%), and 27% (and 63%) increases relative to the control strain 5D, respectively (Table S4). Under other conditions, there were no significant differences between the growth of the mutant and control strains. Therefore, the inclusion of glycerol significantly enhanced the tolerance of the mutant strain to high 2-PE, sugar, and salt stresses, with the Stl1^F427L^ mutation facilitating tolerance to high 2-PE concentrations. These findings are consistent with adaptive evolution results observed in the laboratory (2), as well as high salt and high sugar stresses.

### The increase in external glycerol intake associated with the Stl1^F427L^ mutation

**Fig 3:**
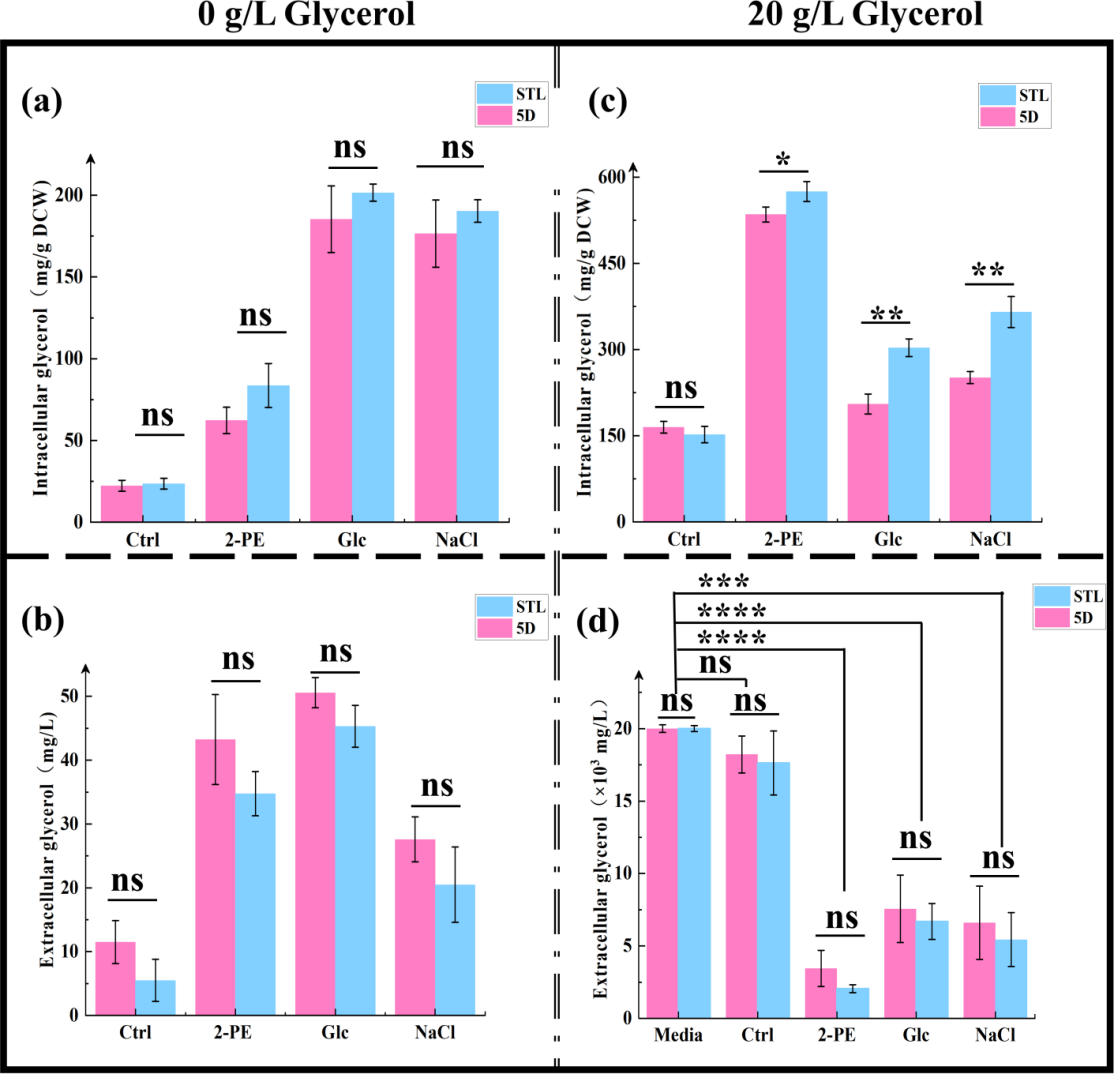
Comparison of intracellular and extracellular glycerol content in the control strain 5D and mutant strain STL. (a–d) Glycerol content in (a) cell extracts and (b) conditioned media without glycerol, as well as (c) cell extracts and (d) conditioned media with 20 g/L glycerol, from cultures of the control and mutant strains, quantified using a glycerol kit: *P < 0.05; **P < 0.01; ***P < 0.001; ****P < 0.0001; ns, not significant (one-way ANOVA with Tukey’s multiple comparison). Media group comprised the YPD medium without microorganism culture; Ctrl group was cultured in the YPD medium; 2-PE group was cultured in 3.0 g/L 2-PE–YPD medium; Glc group was cultured in 700 g/L glucose–YPD medium; and NaCl group was cultured in 60 g/L NaCl–YPD medium.

Given the pronounced impact of glycerol on stress tolerance in the STL strain, we explored the underlying causes by examining changes in intracellular and extracellular glycerol levels in the control strain 5D and mutant strain STL under various culture conditions. Intracellular and extracellular glycerol content differences are shown in Fig. 3. Following the addition of glycerol, under 3.0 g/L 2-PE, 60 g/L NaCl, and 700 g/L glucose stresses, significant differences in intracellular glycerol content were observed between the control and mutant strains. However, there were no significant differences in extracellular glycerol content between the two strains. Notably, both strains exhibited significantly decreased extracellular glycerol content compared with the media-only group. Under 3.0 g/L 2-PE, 700 g/L glucose, and 60 g/L NaCl stresses, the intracellular glycerol content of mutant strain STL was 575.03 ± 17.42, 303.32 ± 15.32, and 365.32 ± 26.91 mg/g DCW, representing 1.07-, 1.47-, and 1.45-fold increases relative to control strain 5D, respectively. In the absence of glycerol, no significant change in intracellular or extracellular glycerol content was observed in either the control or mutant strains. Additionally, under various stress pressures, in the absence of glycerol, both strains exhibited more concave centers, resembling the characteristic “doughnut” morphology typical of yeast under stress. Yan et al. (53) observed similar morphology in their study on the resistance of *Zygosaccharomyces rouxii* to high-temperature stress. Upon glycerol addition, cells of the mutant strain STL appeared significantly fuller than those of the control strain 5D. However, the number of cells with “doughnut” characteristics did not differ significantly between the two strains, and the degree of central depression was lower than that observed without glycerol addition (Fig. S4).

**Fig 4:**
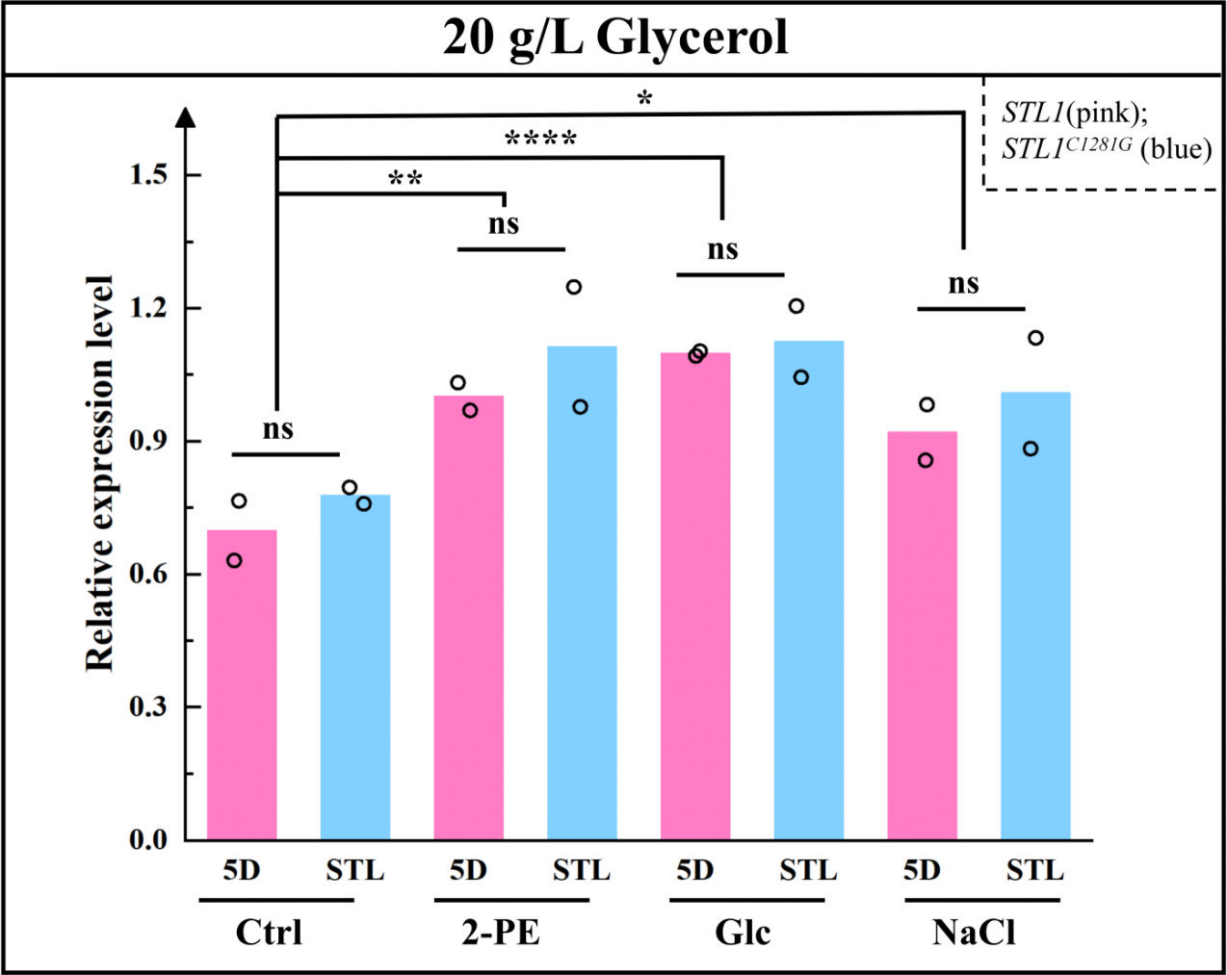
Comparison of the expression levels in the control strain 5D and mutant strain STL addition 20 g/L glycerol. *P < 0.05; **P < 0.01; ****P < 0.0001; ns, not significant (one-way ANOVA with Tukey’s multiple comparison). Media group comprised the YPD medium without microorganism culture; Ctrl group was cultured in the YPD medium; 2-PE group was cultured in 3.0 g/L 2-PE–YPD medium; Glc group was cultured in 700 g/L glucose–YPD medium; and NaCl group was cultured in 60 g/L NaCl–YPD medium.

qPCR results (Fig. 4) indicated that after glycerol addition, no significant change occurred in the expression levels of *STL1* and *STL1^C1281G^* in 3.0 g/L 2-PE, 700 g/L glucose, and 60 g/L NaCl stress medium. However, compared with the nonstressed group, the expression levels of *STL1* and *STL1^C1281G^* increased significantly under different stress conditions. In summary, the *STL1* mutation had no effect on expression levels, whereas expression levels of *STL1* in the control strain 5D and *STL1^C1281G^* in the mutant strain STL significantly increased under stress stimulation. Therefore, the possibility that enhanced glycerol transport capacity is due to a higher number of transporters was excluded.

Under identical stress conditions, glycerol synthesis and efflux remained consistent without glycerol addition. However, once glycerol was added, glycerol synthesis and excretion levels were expected to remain similar to those in the absence of glycerol under the same stress conditions. Nevertheless, the intracellular glycerol content of the mutant strain STL was significantly higher than that of the control strain 5D, and the change in extracellular glycerol content was slightly higher in the mutant than in the control. Combined with the logarithmic growth curve and morphological observations, OD_600 nm_ and cell morphology did not differ significantly between the two strains. We speculate that more glycerol is transported by the Stl1^F427L^ protein per unit of yeast cells, indicating that the Stl1 protein (F427L) mutation is conducive to extracellular glycerol uptake, significantly increases intracellular glycerol content, alleviates osmotic pressure under high salt and high sugar conditions, and may relieve 2-PE stress and enhance cell tolerance to stress. Simultaneously, under different stress conditions for glycerol, the intracellular glycerol content increased significantly, which also reflects that the mutation of the Stl1 protein may affect the transport of glycerol.

### Influence of glycerol intake on ROS levels under stress conditions

**Fig 5:**
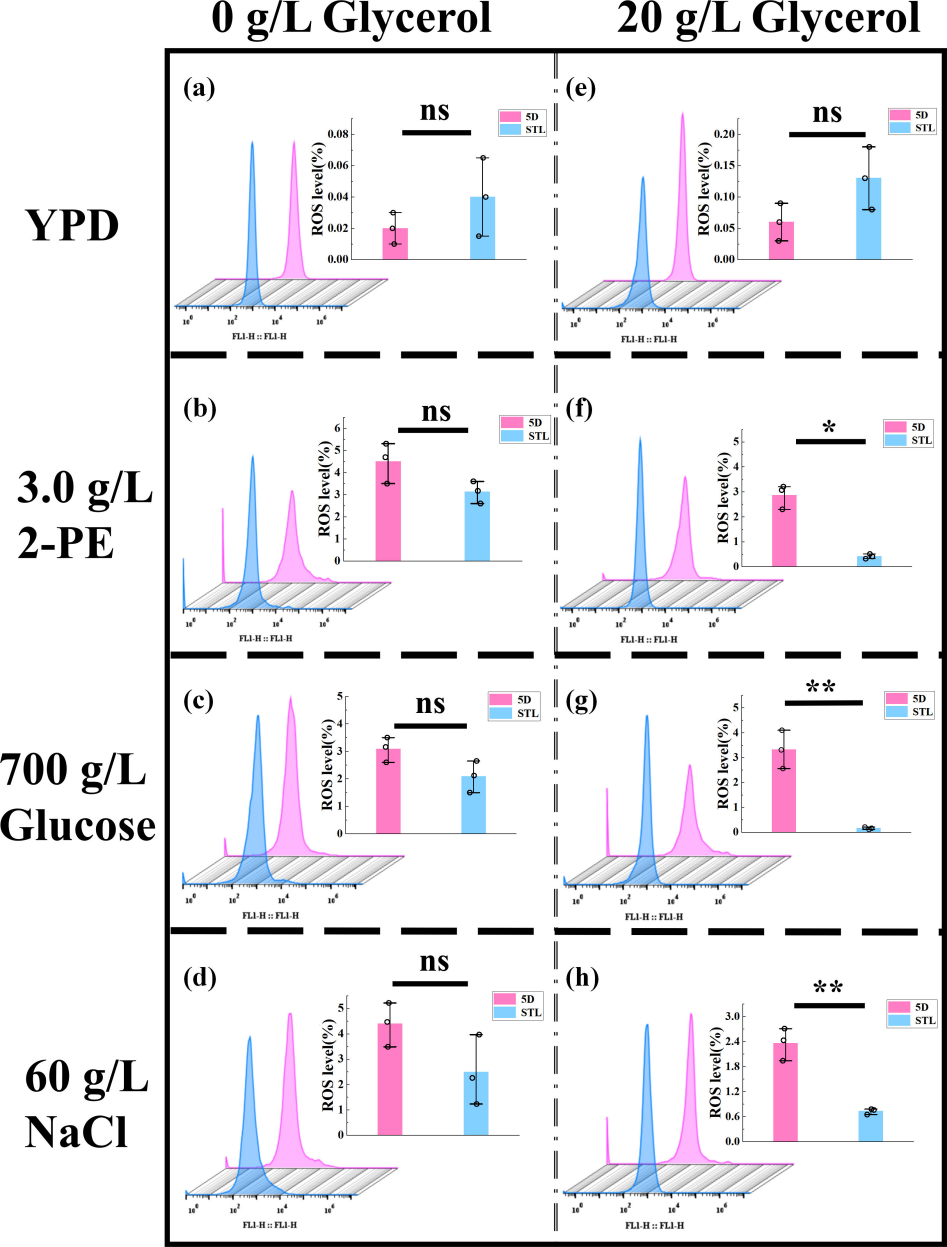
Comparison of intracellular ROS levels in the control strain 5D and mutant strain STL. (**a and e**) ROS levels of (**a**) 0 g/L glycerol and (**e**) 20 g/L glycerol in YPD media from cultures of the control and mutant strains: ns, not significant (one-way ANOVA). (**b and f**) ROS levels of (**b**) 0 g/L glycerol and (**f**) 20 g/L glycerol in YPD media with 3.0 g/L 2-PE from cultures of the control and mutant strains: *P < 0.05; ns, not significant (one-way ANOVA). (**c and g**) ROS levels of (**c**) 0 g/L glycerol and (**g**) 20 g/L glycerol in YPD media with 700 g/L glucose from cultures of the control and mutant strains: **P < 0.01; ns, not significant (one-way ANOVA). (**d and h**) ROS levels of (**d**) 0 g/L glycerol and (**h**) 20 g/L glycerol in YPD media with 60 g/L NaCl from cultures of the control and mutant strains: **P < 0.01; ns, not significant (one-way ANOVA). In all cases, ROS levels were quantified using a flow cytometer.

When yeast cells are subjected to external 2-PE (54), high-sugar (55), and high-salt (56) stresses, rapid accumulation of ROS occurs within the cells (57). ROS, including superoxide anions, hydrogen peroxide, hydroxyl radicals, ozone, and singlet oxygen, cause intracellular oxidative stress, resulting in intracellular protein denaturation (11, 58), DNA damage (59–61), and cell membrane degradation through the destruction of the phosphatidic acid molecular layer (62), ultimately inhibiting cell growth (63). Changes in ROS levels in yeast cells can indicate alterations in cell tolerance to external stressors. Intracellular ROS detection (Fig. 5) revealed that after the addition of 20 g/L glycerol, significant differences (*P* < 0.05) in intracellular ROS levels were observed between both the control strain 5D and mutant strain STL under 3.0 g/L 2-PE, 700 g/L glucose, and 60 g/L NaCl stresses. The mutant strain exhibited ROS levels of 0.41%, 0.16%, and 0.73%, representing 85.66%, 95.19%, and 69.07% decreases relative to the control strain (2.86%, 3.32%, and 2.36%), respectively. Under other conditions, no significant changes were observed. These results suggest that increased glycerol intake can significantly reduce intracellular ROS levels in yeast. Consequently, the mutant strain exhibits significantly enhanced tolerance to 2-PE, high sugar, and high salt stresses.

### Influence of glycerol intake on cell membrane composition under stress conditions

**Fig 6:**
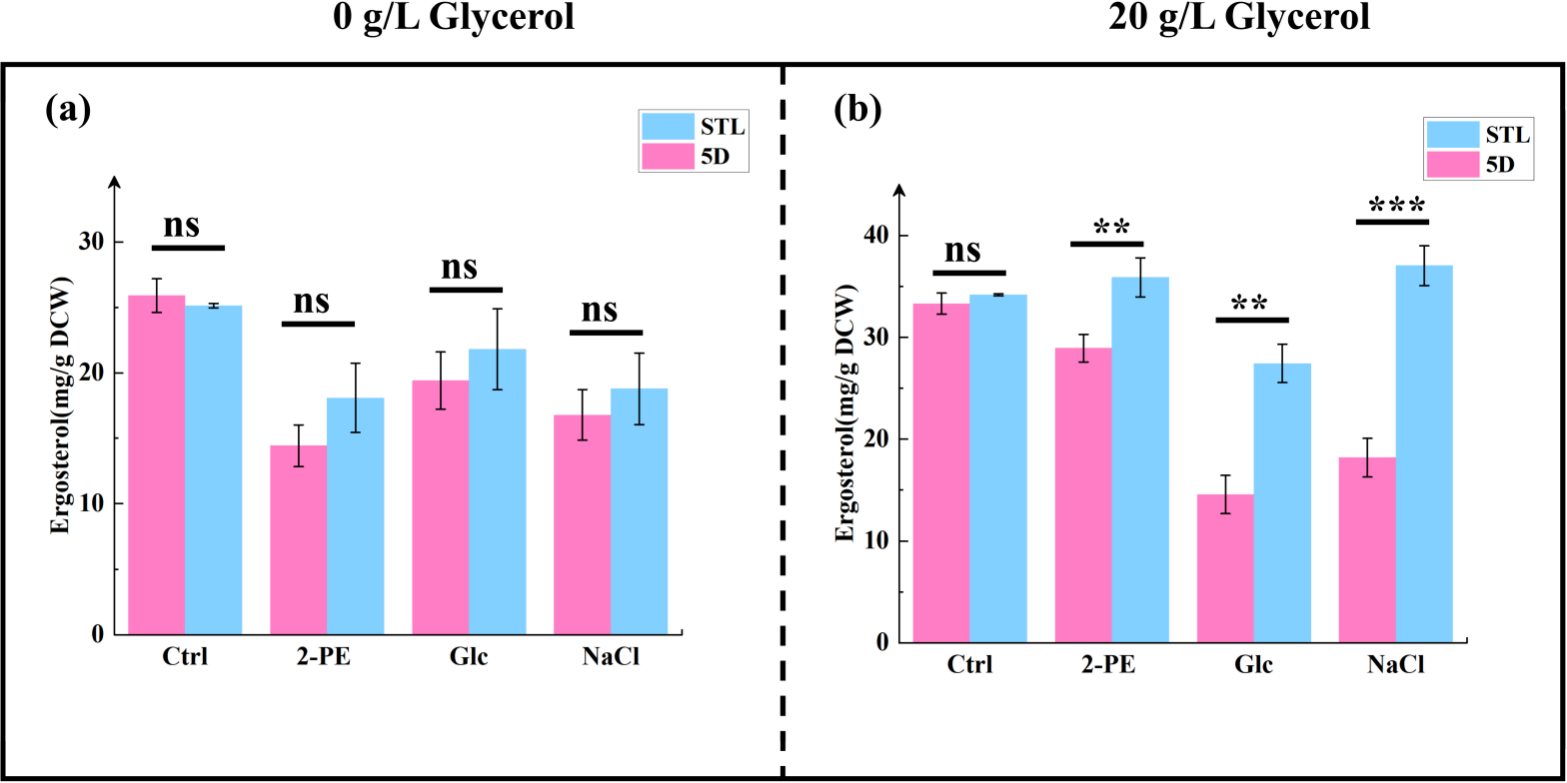
Comparison of intracellular ergosterol content in the control strain 5D and mutant strain STL. (**a and b**) Ergosterol content of cell extracts (**a**) without glycerol and (**b**) with 20 g/L glycerol from cultures of the control and mutant strains, quantified using an ultraviolet spectrophotometer. *P < 0.05; **P < 0.01; ***P < 0.001; ns, not significant (one-way ANOVA). Ctrl group was cultured in the YPD medium; 2-PE group was cultured in 3.0 g/L 2-PE–YPD medium; Glc group was cultured in 700 g/L glucose–YPD medium; and NaCl group was cultured in 60 g/L NaCl–YPD medium.

Yeast cells subjected to external stresses, such as 2-PE, high sugar, and high salt levels, face challenges in terms of their cell membranes and cell walls. In response to stress, yeast cells synthesize ergosterol (2, 9, 17, 64, 65) to repair and reinforce damaged cell membranes and walls. This adaptive response enhances stress tolerance in yeast. Yeast cells also adjust the types and content of unsaturated fatty acids (UFAs) and saturated fatty acids (SFAs) in response to environmental stressors (66–68). Ergosterol test results are shown in Fig. 6. After the addition of 20 g/L glycerol, significant differences in intracellular total ergosterol content were observed between the control strain 5D and the mutant strain STL under 3.0 g/L 2-PE, 60 g/L NaCl, and 700 g/L glucose stresses, with total ergosterol content in the mutant strain recorded at 35.73 ± 1.92, 37.03 ± 1.95, and 27.43 ± 1.88 mg/g DCW, representing 1.24-, 2.04-, and 1.88-fold increases relative to the control strain, respectively. Under other conditions, no significant differences in total ergosterol content were observed between the two strains.

**Fig 7:**
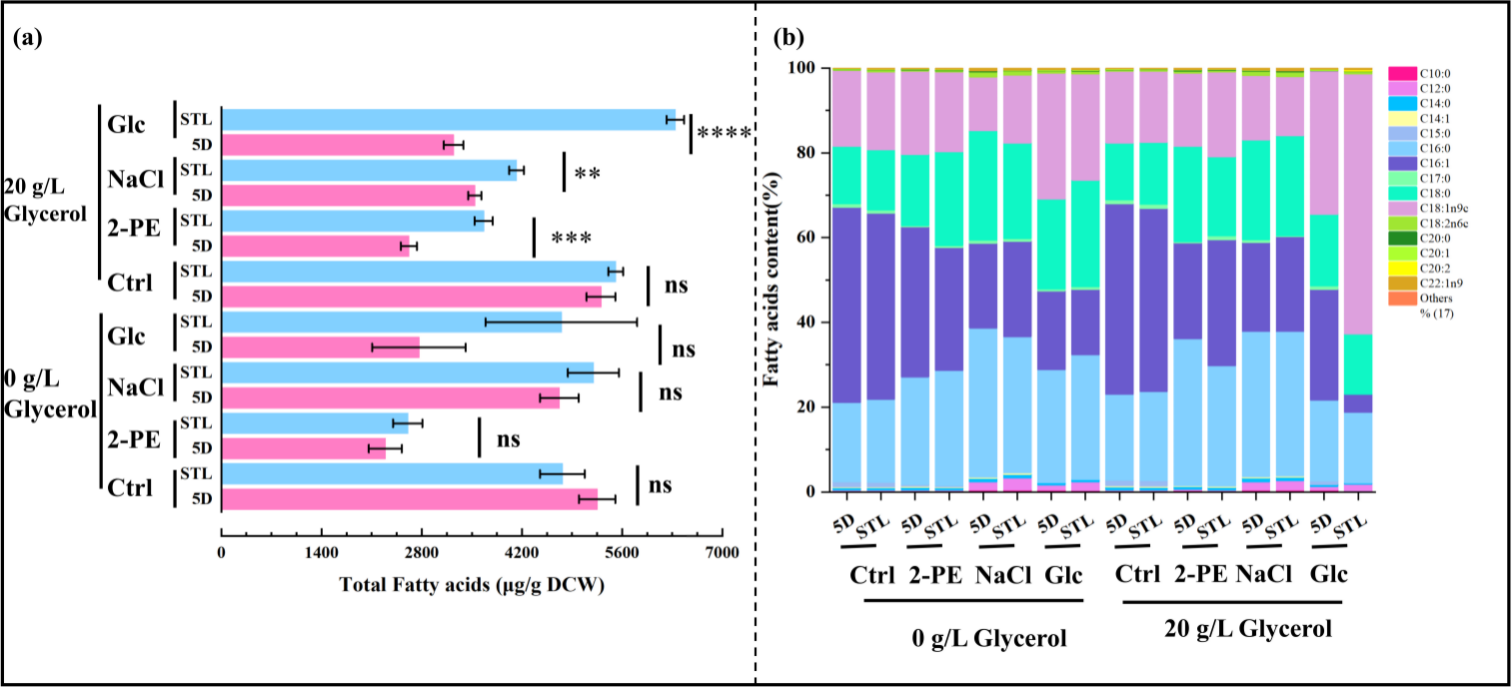
Comparison of the intracellular fatty acid content and USF/SFA ratio of the control strain 5D and mutant strain STL. (**a**) Intracellular fatty acid content of 0 g/L glycerol in YPD, 3.0 g/L 2-PE–YPD, 60 g/L NaCl–YPD, and 700 g/L glucose–YPD media, and 20 g/L glycerol in YPD, 3.0 g/L 2-PE–YPD, 60 g/L NaCl–YPD, and 700 g/L glucose–YPD media from cultures of the control and mutant strains, quantified using gas chromatography.*P < 0.05; **P < 0.01; ***P < 0.001; ****P < 0.0001; ns, not significant (one-way ANOVA ) (**b**) Fatty acid content of the control strain 5D and mutant strain STL (%). Abbreviations: capric acid (C_10:0_), lauric acid (C_12:0_), myristic acid (C_14:0_), pentadecanoic acid (C_15:0_), palmitic acid (C_16:0_), palmitoleic acid (C_16:1_), seventeen carbonic acid (C_17:0_), stearic acid (C_18:0_), oleic acid (C_18:1n9c_), linoleic acid (C_18:2n6c_), arachidic acid (C_20:0_), arachidonic acid (C_20:1_), 11,14-eicosadienoic acid (C_20:2_), erucic acid (C_22:1n9_), and lignoceric acid (C_24:0_).

Differences in total fatty acid content and the percentage contents of various fatty acids are shown in Fig. 7. The total fatty acid content in the mutant strain (4.76 mg/g DCW) was 91.23% of that observed in the control strain (5.21 mg/g DCW) in the absence of glycerol. However, following glycerol addition, the total fatty acid content in the mutant (5.51 mg/g DCW) was 1.04-fold higher than that in the control (5.30 mg/g DCW). Under 2-PE, high sugar, and high salt stresses, the total fatty acid content was higher in the mutant strain STL than in the control strain 5D, regardless of the presence of glycerol. However, when glycerol was added under 2-PE, high sugar, and high salt stress conditions, total fatty acid levels increased by 1.40-, 1.16-, and 1.95-fold compared with the absence of glycerol. The percentage contents of various fatty acids remained largely unchanged in both the control strain 5D and mutant strain STL, regardless of glycerol addition.

The percentage content of palmitoleic acid (C_16:1_) was consistently high at 46.00% and 46.05% in the control and mutant, respectively. In the absence of glycerol, the content of palmitoleic acid (C_16:1_) in the mutant strain exposed to 2-PE and high sugar stresses decreased by 18.03% and 17.07%, respectively, compared with that in the control strain, whereas the percentage content of stearic acid (C_18:0_) increased by 31.40% and 18.40%, respectively. When glycerol was added, the palmitoleic acid (C_16:1_) content in the mutant strain under 2-PE and high salt stresses increased by 31.52% and 6.62%, respectively, compared with the control strain. Under high sugar stress, the percentage of palmitoleic acid (C_16:1_) in the mutant strain decreased to 16.04% of that in the control strain, whereas the percentage of oleic acid (C_18:1n9c_) significantly increased, reaching 1.81-fold the level observed in the control strain. Additionally, under 2-PE, high salt, and high sugar conditions, the stearic acid (C_18:0_) content of the mutant strain decreased by 17.20%, 0.43%, and 15.65% compared with the control strain, respectively.

**Fig 8:**
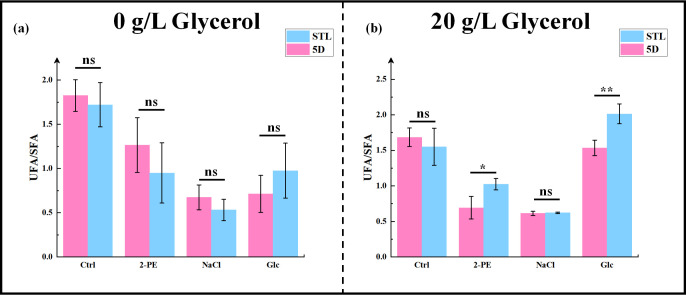
Comparison of the intracellular USF/SFA ratio (**a and b**) of the control strain 5D and mutant strain STL. USF/SFA ratio of (**a**) 0 g/L glycerol and (**b**) 20 g/L glycerol in YPD, 3.0 g/L 2-PE–YPD, 60 g/L NaCl–YPD, and 700 g/L glucose–YPD media from cultures of the control and mutant strains. *P < 0.05; **P < 0.01; ns, not significant (one-way ANOVA).

Regarding the UFA/SFA ratio (Fig. 8), no significant difference was observed between the control strain 5D and the mutant strain STL under nonstress conditions, regardless of the presence of glycerol. However, with 2-PE and high sugar stress, the UFA/SFA ratio was 1.48- and 1.31-fold higher, respectively, in the mutant than in the control. Notably, it was observed that increased glycerol consumption led to higher levels of ergosterol and fatty acids, consistent with expectations. These findings suggest that enhanced glycerol intake can reinforce the robustness of *S. cerevisiae* cell membranes.

### Potential mechanism underlying the changes in physicochemical properties of yeast under multiple stress with the addition of glycerol after the Stl1 protein mutation

Upon glycerol addition, the glycerol transport capacity, ergosterol production capacity, and total fatty acid content of the mutant strain STL increased significantly. Whether this correlates with a substantial reduction in intracellular ROS levels warrants further investigation.

To explore the internal relationship of the abovementioned intracellular environmental changes, we aimed to link acetyl-CoA generated via pyruvate metabolism with the alterations observed in ergosterol and fatty acids. Pyruvate, as a central metabolic product, may be associated with glycerol as the secondary carbon source entering the central metabolic pathway. Pyruvate synthesis (69) is integral to central carbon metabolism. Both the mutant strain STL and the control strain 5D exhibited identical intracellular glucose content under the same culture conditions. Therefore, the variation in pyruvate content could indicate glycerol’s potential entry into the central carbon metabolism (70). When subjected to external stress, *S. cerevisiae* synthesizes ergosterol and fatty acids via acetyl-CoA in the central metabolic pathway to enhance cell membrane resilience and resist external stress. Ergosterol, a cholesterol-like compound, plays a pivotal role in fungal cell membranes (71). It regulates cell membrane fluidity and permeability, influencing membrane-binding protein function (72). Additionally, ergosterol (73) serves as a signaling molecule, transmitting pressure signals and enhancing membrane-bound enzyme activity to reduce ROS levels. Therefore, in the present study, the intracellular glucose and pyruvate contents as well as the CAT and SOD activities of the mutant strain STL and the control strain 5D were compared under different stresses with glycerol addition.

**Fig 9:**
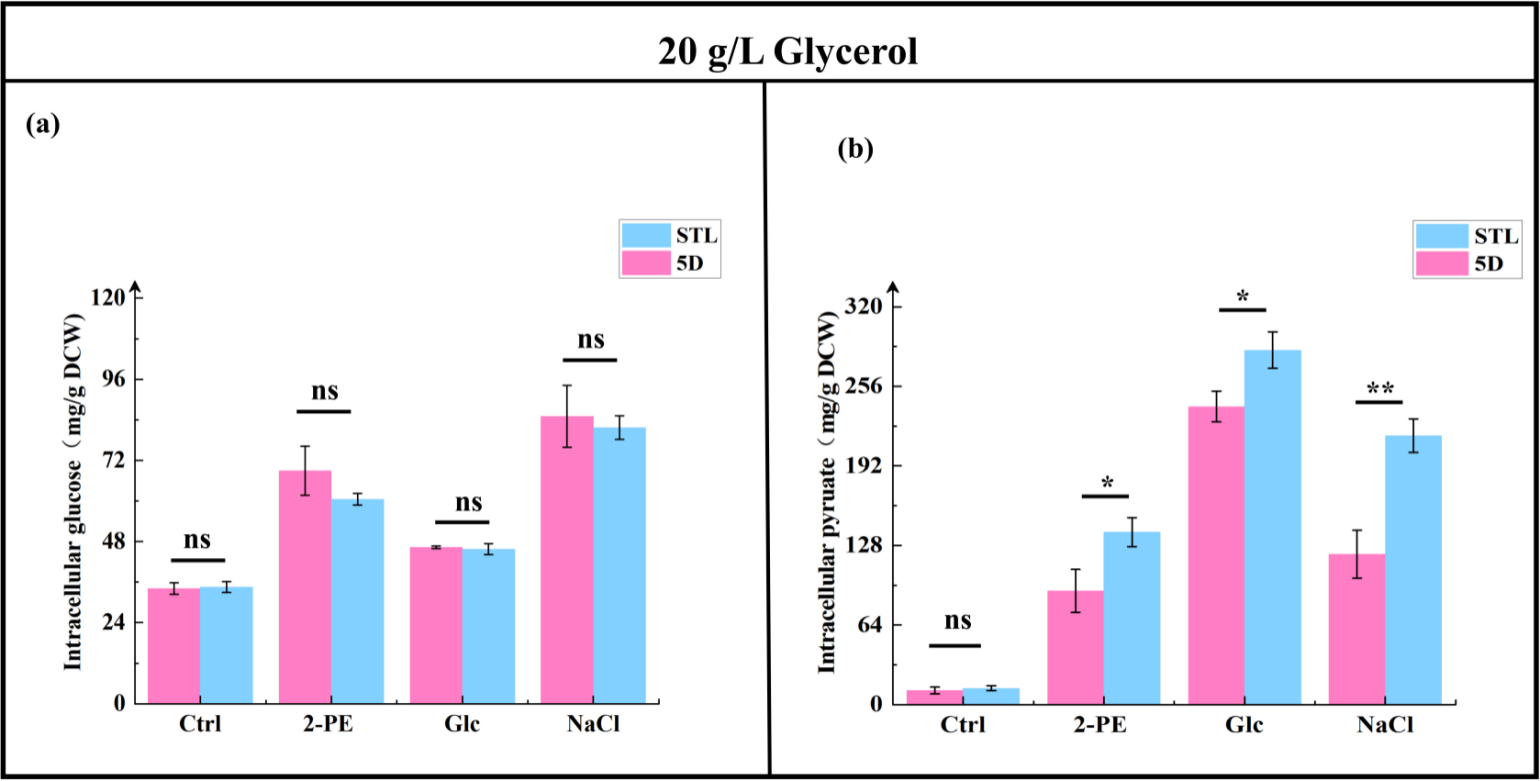
Comparison of the intracellular glucose (**a**) and pyruvate contents (**b**) in the control strain 5D and mutant strain STL with 20 g/L glycerol addition. *P < 0.05; **P < 0.01; ns, not significant (one-way ANOVA). Ctrl group was cultured in the YPD medium; 2-PE group was cultured in 3.0 g/L 2-PE–YPD medium; Glc group was cultured in 700 g/L glucose–YPD medium; and NaCl group was cultured in 60 g/L NaCl–YPD medium.

**Fig 10:**
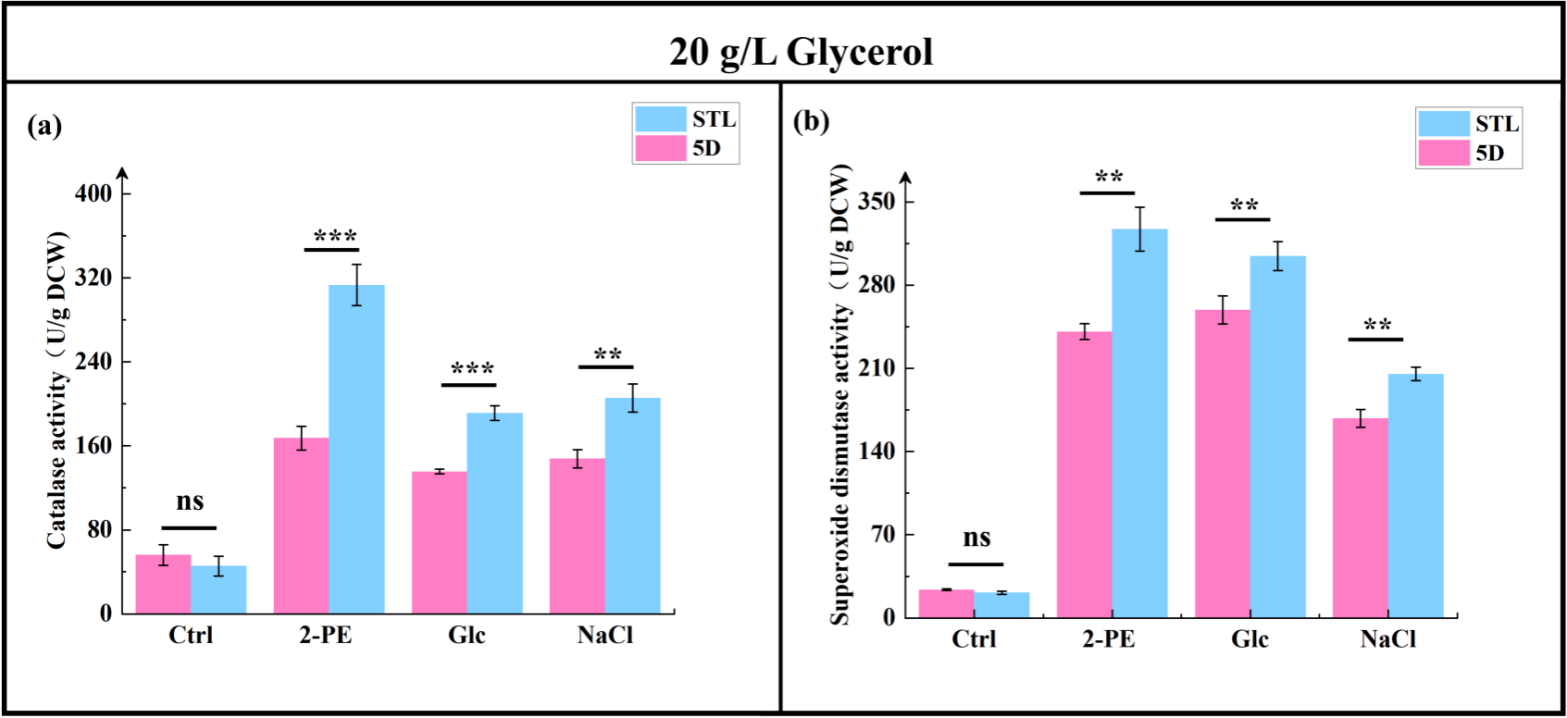
Comparison of the catalase (**a**) and superoxide dismutase (**b**) activity in the control strain 5D and mutant strain STL addition 20 g/L glycerol. *P < 0.05; **P < 0.01; ***P < 0.001; ns, not significant (one-way ANOVA). Ctrl group was cultured in the YPD medium; 2-PE group was cultured in 3.0 g/L 2-PE–YPD medium; Glc group was cultured in 700 g/L glucose–YPD medium; and NaCl group was cultured in 60 g/L NaCl–YPD medium.

The results of intracellular glucose and pyruvate content analysis (Fig. 9) revealed no significant difference in glucose levels between the control and mutant strains under 3.0 g/L 2-PE, 700 g/L glucose, and 60 g/L NaCl stresses; however, the mutant strain STL exhibited 51.71%, 18.93%, and 78.85% increases in pyruvate content (138.77 ± 11.72, 285.16 ± 14.63, and 216.37 ± 13.50 mg/g DCW), respectively, compared with the control strain 5D (91.47 ± 17.24, 239.78 ± 12.37, and 120.98 ± 19.16 mg/g DCW, respectively). Results of CAT and SOD activities (Fig. 10) showed that under 3.0 g/L 2-PE, 700 g/L glucose, and 60 g/L NaCl stresses, the mutant strain STL’s CAT activity increased by 87.13%, 41.35%, and 39.14% (313.13 ± 19.65, 191.32 ± 6.95, and 205.56 ± 13.50 U/g DCW), respectively, compared with the control strain 5D’s activity (167.33 ± 11.39, 135.53 ± 2.19, and 147.74 ± 8.63 U/g DCW, respectively). Similarly, the SOD activity of the mutant strain STL increased by 35.82%, 17.46%, and 22.17% (327.10 ± 18.42, 304.33 ± 12.10, and 205.24 ± 5.67 U/g DCW), respectively, compared with the control strain 5D (240.84 ± 16.69, 259.10 ± 11.87, and 168.00 ± 7.49 U/g DCW, respectively).

Under stress conditions, glucose’s effect weakens and *S. cerevisiae* activates exogenously added glycerol as a spare carbon source. This allows the mutant strain STL to import more glycerol from the environment into central carbon metabolism for pyruvate synthesis. Subsequently, synthesized pyruvate is converted into acetyl-CoA, providing raw materials for ergosterol and fatty acid synthesis. Consequently, ergosterol and fatty acid synthesis increase significantly in the mutant strain STL under various stress pressures after glycerol addition. Moreover, ergosterol as a signal molecule led to increased activities of CAT and SOD attached to cell membranes or organelles, resulting in significantly reduced intracellular ROS levels in the mutant strain STL under different stresses in the glycerol addition group.

### Bioinformatics analysis of transporter Stl1 and mutant Stl1^F427L^ structures and glycerol binding abilities

To further explore the enhanced glycerol transport capacity exhibited by the mutant strain STL, we applied bioinformatics techniques to investigate various aspects of protein structure, protein–small molecule complexes, and molecular dynamics simulations. In protein property analysis, the hydrophilic index, reflecting the average hydrophilicity of a protein, and the hydrophilic and hydrophobic fractions of individual amino acids can provide insights into a protein’s hydrophilic and hydrophobic characteristics (74). Larger hydrophilic indices and mean hydrophilicity values indicate higher hydrophobicity and lower hydrophilicity. Negative scores correspond to hydrophilic amino acids, whereas positive scores indicate hydrophobic amino acids.

The FASTA-format sequences of the glycerol/H^+^ transporter protein Stl1 and its mutant protein Stl1^F427L^ were used to evaluate physicochemical parameters. The molecular formula of Stl1 is C_2896_H_4423_N_737_O_827_S_24_, with a relative molecular mass of 63565.86 and a theoretical isoelectric point of 6.13. Stl1 comprises 569 amino acids, with leucine being the most abundant at 9.1%, followed by glycine, phenylalanine, and serine, each accounting for 8.1%. The number of negatively (Asp + Glu) and positively (Arg + Lys) charged residues is 53 and 49, respectively, and its instability index of 29.04 (<40) indicates protein stability. Its hydrophilic index is 87.10, with an average hydrophilic value of 0.093. The molecular formula of Stl1^F427L^ is C_2893_H_4425_N_737_O_827_S_24_, with a relative molecular mass of 63531.84 and a theoretical isoelectric point of 6.13. Akin to Stl1, Stl1^F427L^ comprises 569 amino acids, with leucine being the most abundant at 9.1%, followed by serine and glycine at 8.1%. Its number of negatively (Asp + Glu) and positively (Arg + Lys) charged residues is identical to Stl1, as is its instability index (indicating protein stability). However, the hydrophilic index of Stl1^F427L^ is 87.79, with an average hydrophilic value of 0.095. The physical and chemical properties of Stl1 and Stl1^F427L^ are detailed in Table S5. Hydrophilic indices and average hydrophilic values, along with single amino acid hydropathy scores, reflect protein water-based characteristics. The hydrophilic index and average hydrophilic value indicate overall water interaction, where a higher positive hydrophilic index suggests greater hydrophobicity. Single amino acid hydropathy scores provide specific site information: negative scores indicate hydrophilic amino acids, whereas other scores denote hydrophobic amino acids. Therefore, based on the abovementioned findings of protein analysis, Stl1 and Stl1^F427L^ are considered stable, acidic, and hydrophobic proteins.

Protein hydrophobicity analysis of Stl1 and Stl1^F427L^ (Fig. S5) revealed distinct hydrophobic and hydrophilic regions. Notably, the residue 214 had the highest hydrophobicity of 3.256, whereas the lowest value of −3.244 was observed at residue 70. Residue 427 of Stl1 and Stl1^F427L^ had hydrophobicity values of −0.011 and 0.100, respectively. These findings suggest that both Stl1 and Stl1^F427L^ are hydrophobic proteins.

The results of secondary structure prediction showed that the Stl1 and Stl1^F427L^ proteins comprise α-helix, β-corner, extended chain, and random curl; of these, the proportion of α-helix in Stl1^F427L^ is 14.58% higher than that in Stl1, whereas that of β-corner, extended chain, and random curl in Stl1^F427L^ is 9.49%, 17.25%, and 6.58% lower than that in Stl1, respectively. The secondary structure data of the Stl1 and Stl1^F427L^ proteins are shown in Table S6 and Fig. S6. The TMHMM prediction revealed that both the Stl1 and Stl1^F427L^ proteins were nearly identical in their transmembrane domains (Fig. S7), each comprising 12 transmembrane domains. Prediction of the tertiary structure of Stl1 and Stl1^F427L^ proteins (Fig. S8) showed that the trust value of the Stl1 protein model C score was −1.34 and the TM score was 0.55 ± 0.15, whereas the Stl1^F427L^ protein model C score was −1.10 and TM score was 0.58 ± 0.14. Both the Stl1 and Stl1^F427L^ protein tertiary structure models have good reliability and correct topology. The spatial filling structure prediction of Stl1 and Stl1^F427L^ (Fig. S9) showed that compared with Stl1, the mutant protein Stl1^F427L^ had no significant difference in the spatial structure inside and outside the cell membrane. Interestingly, it exhibited signs of charge rearrangement, but the relationship between its charge changes and the mutant protein’s function requires further investigation.

The Ramachandran plot (Fig. S10) revealed that the residues in most favored regions for Stl1^F427L^ and Stl1 proteins was 91.9%, except for the difference in the number of individual amino acids (F and L). The model is reliable and can be used for molecular docking and molecular dynamics simulation.

**Fig 11:**
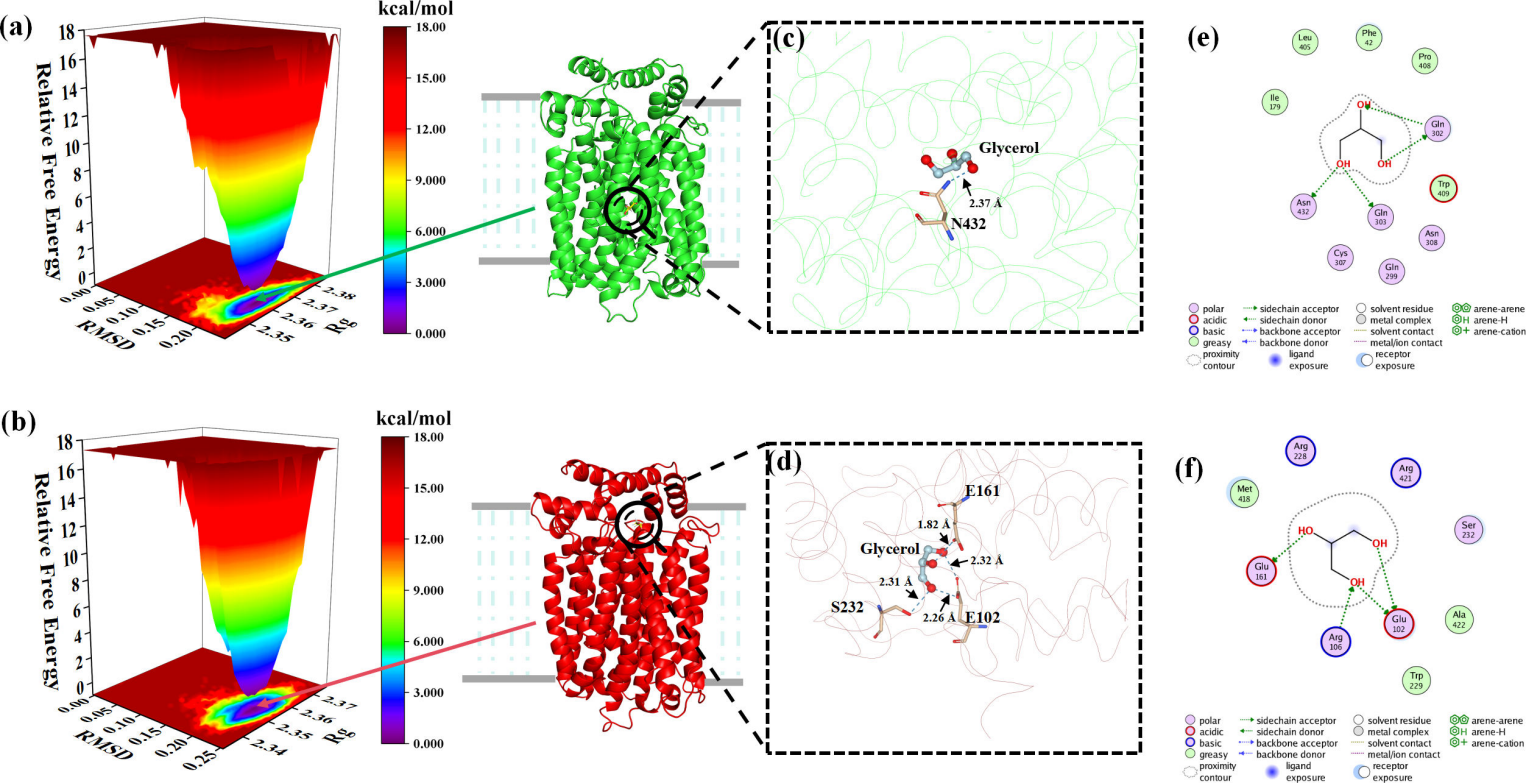
Molecular docking and molecular dynamics simulation of *S. cerevisiae* Stl1 and Stl1^F427L^ proteins in relation to glycerol. (**a and b**) Minimum energy complex structure and corresponding free energy landscape diagram of (**a**) Stl1 (the 180,820th frame) and (**b**) Stl1^F427L^ (the 150,100th frame) in relation to glycerol. The typical snaps from corresponding minimum energy wells were extracted. Gray lines denote the phospholipid bilayers of cell membranes, and the dashed blue line area represents the transmembrane region. (**c and d**) Complex-binding pockets in trace representations, showing the interaction of ligands with receptors through hydrogen bonds in the complexes of (**c**) Stl1 and (**d**) Stl1^F427L^ with glycerol. (**e and f**) Two-dimensional diagram of protein–small molecule interactions in the complex-binding pockets of (**e**) Stl1 and (**f**) Stl1^F427L^ in relation to glycerol.

Molecular docking showed that the binding sites of Stl1^F427L^ and glycerol were closer to the cytoplasm than Stl1, and there were significant differences in the structure of the transmembrane region near the cytoplasm between the two models, mainly reflected in the spatial structure changes of the loop ring and α-helix (Fig. 11a and b). Simultaneously, Stl1^F427L^–glycerol complex formed more hydrogen bonds with glycerol (four hydrogen bonds) compared to the Stl1–glycerol complex (one hydrogen bond). In the complex formed by Stl1 protein and glycerol, the hydrogen bond distance between the glycerol molecule and N432 of Stl1 was 2.37 Å. In the complex formed by the Stl1^F427L^ protein and glycerol, the hydrogen bond distances between glycerol and Stl1^F427L^ were 1.79 Å at E161, 2.26 Å and 2.32 Å at E102, and 2.31 Å at S232, respectively.

To determine the degree and stability of glycerol molecule binding to Stl1^F427L^ and Stl1 proteins, we performed a 200-ns molecular dynamics simulation to analyze the docking results. The MD simulation results are shown in Fig. S10.

The RMSD serves as an index to assess protein–receptor complex stability. A smoother RMSD curve indicates a more stable complex formation (75). As shown in Fig. S11a, the fluctuation range of the RMSD curves of the Stl1–glycerol and Stl1^F427L^–glycerol complexes was within 1 nm throughout the whole process. There were no large fluctuations, and the fluctuation range was approximately 0.2 nm, indicating that the Stl1–glycerol and Stl1^F427L^–glycerol complexes were stable from the perspective of RMSD.

The RMSF curve reflects the fluctuation degree of amino acid residues in the protein during kinetic simulation. Higher RMSF values signify greater residue fluctuations, and vice versa (76). As shown in Fig. S11b, the RMSF curves of the Stl1 and Stl1^F427L^ protein–glycerol complexes fluctuated within a 0.6 nm range, although the Stl1^F427L^ protein–glycerol complex showed less fluctuation, indicating greater stability.

Rg characterizes the compactness and stability of the structure. A larger Rg indicates severe expansion during dynamic simulation, whereas a smaller Rg indicates compactness and stability (77). As shown in Fig. S11c, the Stl1–glycerol and Stl1^F427L^ protein–glycerol complex remained stable after 20 ns, with a range of 2.35–2.40 nm observed. However, the Stl1^F427L^–glycerol complex had a slightly lower Rg than the Stl1–glycerol complex after stabilizing. These findings suggest that Stl1^F427L^ forms a compact and stable complex with glycerol, whereas the Stl1–glycerol complex has less overall compactness and stability.

To investigate the hydrogen bond properties of the Stl1–glycerol and Stl1^F427L^–glycerol complexes, the hydrogen bonds between glycerol and Stl1 or Stl1^F427L^ were quantified (78). As shown in Fig. S11d, the hydrogen bond number of the Stl1^F427L^–glycerol and Stl1–glycerol complexes generally remained at ≥2 throughout the process, with a maximum of 6 and 8 hydrogen bonds observed, respectively. These results indicate robust hydrophobic interactions between glycerol and both Stl1^F427L^ and Stl1, resulting in the formation of strong binding complexes.

SASA is one of the factors for assessing protein structure folding and stability. Proteins with stable structures tend to have more stable SASA curves (79). As shown in Fig. S11e, the fluctuation of the SASA curves of the Stl1^F427L^–glycerol and Stl1–glycerol complexes was stable throughout the whole process, and the fluctuation range was approximately 200 nm^2^ without large fluctuations, indicating that both the Stl1^F427L^–glycerol and Stl1–glycerol complexes have good stability.

Furthermore, the free energy landscape map was calculated using the in-built gromac scripts g_sham and xpm2txt.py. The Gibbs relative free energy was computed using the RMSD and Rg values, and a free energy landscape map was plotted with RMSD, Rg, and Gibbs relative free energy on the *X*, *Y*, and *Z* axes, respectively. This visualization illustrates the lowest energy conformations during the entire molecular dynamics simulation of the composite structure. When the interaction between the protein and ligand is weak or unstable, the free energy landscape tends to display multiple rough minimum energy clusters on its surface. Conversely, strong and stable interactions often result in the formation of almost single, smooth energy clusters in the potential energy distribution (80). In Fig. 11a and b, the purple/blue dots reflect the lowest energy values, indicating the most stable structure, whereas the red/yellow dots represent unstable structures. A single minimum energy cluster formed in the free energy distribution diagram of the Stl1–glycerol and Stl1^F427L^–glycerol complexes, and the energy cluster distribution was concentrated, indicating that the Stl1–glycerol and Stl1^F427L^–glycerol complexes have good stability (Fig. 11). The energy clusters in the free energy distribution of the Stl1^F427L^–glycerol complex were more concentrated, indicating that the binding stability of the Stl1^F427L^ protein and glycerol was better (Fig. 11).

Further insight into the intermolecular forces between glycerol and Stl1/Stl1^F427L^ (50) was provided through the detailed molecular docking results shown in Fig. 11, with amino acid residues and interaction forces depicted in two-dimensional graphs. The two-dimensional diagram of protein–small molecule interactions (Fig. 11e and f) illustrates the effective insertion of glycerol into the cavities of Stl1 and Stl1^F427L^, with the major binding sites of glycerol and Stl1 identified as Q302, Q303, and N432 and those of glycerol and Stl1^F427L^ found to be E161, R106, and E102. Consequently, although Stl1–glycerol complexes exhibit minimal variation from Stl1^F427L^–glycerol complexes in terms of form and the number of intermolecular interactions, the latter’s binding sites are located in a larger pocket and are closer to the cytoplasm. Stl1^F427L^ may be more conducive to the binding of glycerol, greatly improving the ability of cells to take up glycerol and increasing the accumulation of intracellular glycerol.

**Fig 12:**
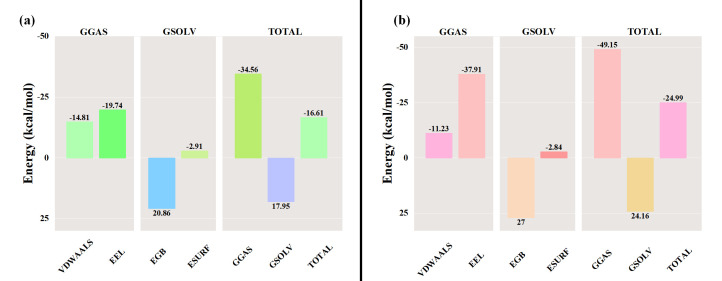
Comparison of MM/PBSA-binding energy of (**a**) the Stl1–glycerol complex and (**b**) the Stl1^F427L^–glycerol complex.

After the Stl1–glycerol and Stl1^F427L^–glycerol complex systems stabilized, the average binding free energy of Stl1 and Stl1^F427L^ to glycerol was calculated using the MM/GBSA method (81). The interactions in the gas phase primarily comprised van der Waals forces and electrostatic interactions. In the MM-PBSA calculations, gas phase Gibbs free energy (GGAS) can be computed as the sum of van der Waals energy (VDWAALS) and electrostatic energy (EEL). For the Stl1–glycerol complex, with VDWAALS at −14.81 kcal/mol and EEL at −19.74 kcal/mol, GGAS was calculated as −34.56 kcal/mol (Fig. 12a). For the Stl1^F427L^–glycerol complex, with VDWAALS at −11.23 kcal/mol and EEL at −37.91 kcal/mol, GGAS was −49.15 kcal/mol (Fig. 12b). Typically, solvent effects are considered via solvent polarization energy (EGB) and surface tension energy (ESURF), which contribute to the solvent Gibbs free energy (GSOLV). For the Stl1–glycerol complex, with EGB at 20.86 kcal/mol and ESURF at −2.91 kcal/mol, GSOLV was 17.95 kcal/mol (Fig. 12a); for the Stl1^F427L^–glycerol complex, with EGB at 27.00 kcal/mol and ESURF at −2.84 kcal/mol, GSOLV was calculated as 24.16 kcal/mol (Fig. 12b). The total free energy (Δ*G*) in the MM-PBSA calculations is the sum of GSOLV and GGAS. Negative Δ*G* values denote stable binding, indicating strong ligand–receptor interactions, whereas positive values suggest instability or repulsion. The MM-PBSA results of the final Stl1- glycerol and Stl1^F427L^–glycerol complexes were −16.61 kcal/mol (Fig. 12a) and −24.99 kcal/mol (Fig. 12b), respectively, indicating that Stl1^F427L^ had a stronger binding affinity with glycerol.

**Fig 13:**
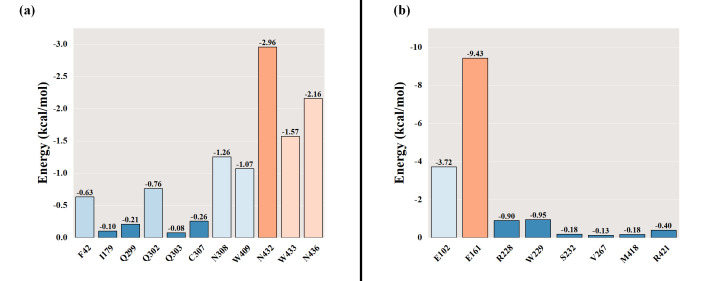
Comparison of the residue-energy analysis of (**a**) the Stl1–glycerol complex and (**b**) the Stl1^F427L^–glycerol complex.

The results of the residue-energy analysis (82) demonstrated that the glycerol ligand binds most effectively to N432 in Stl1 protein, of which the binding energy is −2.96 kcal/mol, indicating that N432 plays a major role in the interaction between glycerol ligand and Stl1 protein (Fig. 13a). Glycerol binds most effectively to E161 in the Stl1^F427L^ protein, of which the binding energy is −9.43 kcal/mol, indicating that E161 plays a major role in the interaction between glycerol ligand and Stl1 protein (Fig. 13b). Moreover, the binding energy of glycerol to E161 of the Stl1^F427L^ protein was lower than that of N432 in Stl1, indicating that Stl1^F427L^ interacts more strongly with glycerol. Thus, Stl1^F427L^ is more conducive to glycerol binding.

**Fig 14:**
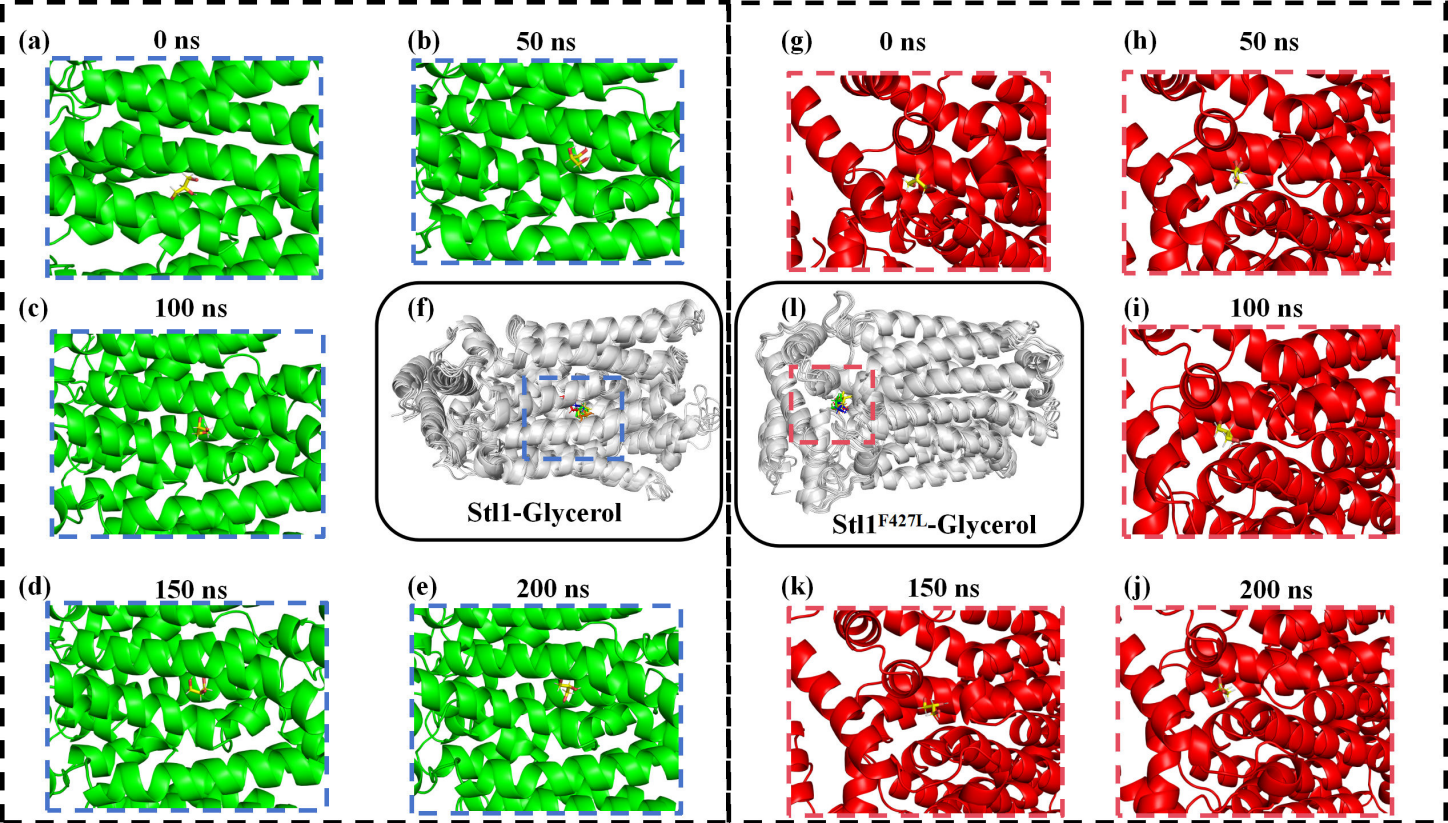
Binding mode of the interactions in molecular dynamics simulations of the Stl1–glycerol and the Stl1^F427L^–glycerol complexes (local amplification). (**a**) Stl1–glycerol complex and (**g**) Stl1^F427L^–glycerol complex at 0 ns; (**b**) Stl1–glycerol complex and (**h**) Stl1^F427L^–glycerol complex at 50 ns; (**c**) Stl1–glycerol complex and (**i**) Stl1^F427L^–glycerol complex at 100 ns; (**d**) Stl1–glycerol complex and (**k**) Stl1^F427L^–glycerol complex at 150 ns; (**e**) Stl1–glycerol complex and (**j**) Stl1^F427L^–glycerol complex at 200 ns; merge of (**f**) Stl1–glycerol complex and (**l**) Stl1^F427L^–glycerol complex at 0, 50, 100, 150, and 200 ns. Moreover, in (**f**) and (**l**), the molecular structures of red, green, blue, yellow, and orange correspond to the five glycerol small molecular structures at 0, 50, 100, 150, and 200 ns, respectively.

To further analyze the binding status of the Stl1–glycerol and Stl1^F427L^–glycerol complexes during the MD simulation process (52), the complex structures at 0, 50, 100, 150, and 200 ns in the MD simulation trajectory of glycerol were extracted and compared. Throughout the MD simulation process, it was evident that glycerol consistently occupies the internal cavity of the active binding site in the Stl1 and Stl1^F427L^ proteins, respectively, without significant changes, indicating that the formed Stl1–glycerol and Stl1^F427L^–glycerol complexes exhibit good stability, as presented in Fig. 14.

## DISCUSSION

In industrial microbial production, chassis cells are inevitably subjected to multiple stresses, including 2-PE, high sugar content, and high salinity. Among these, 2-PE stress poses the most severe threat to microbial cells. Improving yeast’s ability to withstand various stresses is vital for developing robust chassis cells and expanding industrial production (4, 83). Currently, we lack a comprehensive understanding of the mechanisms underlying multistress tolerance, hindering the rational design of stress-tolerant yeast chassis cells. Therefore, in this study, building upon genomic data from previous adaptive laboratory evolution studies, we identified a Stl1 protein mutation (F427L) capable of transporting extracellular glycerol and potentially linked to 2-PE stress tolerance (2). However, the information of the relationship between the Stl1^F427L^ mutant protein and tolerance mechanisms is limited, and the nature of this connection, whether direct or indirect, is not well understood and requires further study.

Yeast cells can activate the HOG signaling pathway under 2-PE stress (10, 17, 84, 85). The sugar transporter-like protein Stl1, found on the cell membrane, is regulated by the Hog1 regulatory factor in the HOG pathway. Stl1 actively transports extracellular glycerol into the cell, accumulating glycerol in response to high sugar and salt levels, a process regulated by *STL1* (86–88). It can be inferred that Hog1, after HOG pathway activation under 2-PE stress, modulates Stl1 expression. The Stl1^F427L^ protein mutation may enhance its affinity for glycerol, increase intracellular glycerol levels, and subsequently affect the cells’ ability to tolerate multiple stresses.

Interestingly, our experimental results indicated that the Stl1^F427L^ protein mutation not only enhanced 2-PE stress tolerance but also significantly enhanced resistance to high sugar and high salt stresses in the mutant strain STL, particularly when glycerol was added. Notably, the best tolerance was observed under high sugar conditions with glycerol, surpassing even the tolerance observed under 2-PE stress. With glycerol supplementation, the mutant strain STL showed a maximum OD_600 nm_ that was 27% higher than that of the control strain 5D and the ratio of OD_600 nm_max is 1.07-fold of that under 2-PE stress. Furthermore, the Stl1^F427L^ mutation significantly increased intracellular glycerol content under glycerol-amended stress environments. By docking mutant transporter Stl1^F427L^ and wild-type protein Stl1 to substrate glycerol and performing molecular dynamics simulations, it was established that Stl1^F427L^ exhibited a higher affinity for small glycerol molecules and might facilitate substrate glycerol uptake. Glycerol accumulation improved not only the strains’ tolerance to hypertonic stress (such as high sugar and high salt stresses) but also tolerance to 2-PE stress in the Stl1^F427L^ mutant in the presence of glycerol. The combination of substrate (glycerol) and membrane protein (Stl1/Stl1^F427L^) via molecular docking and molecular dynamics simulation, along with comprehensive analysis and verification, proved to be reliable. The results are consistent with the related phenomenon of increased glycerol uptake by the mutant strain STL, which may replace the cumbersome purification process, reduce time and cost, and realize the rapid and directed engineering transformation of membrane protein.

Additional experiments showed that after the mutation of the Stl1 protein to Stl1^F427L^, ROS levels in *S. cerevisiae* were significantly reduced in environments with both glycerol and stress. Glycerol, acting as an osmotic balancer and a reducing agent, likely balances intracellular osmotic pressure and regulates metabolism, reducing ROS accumulation (84, 89, 90) and production (91, 92), respectively. Furthermore, cellular stress tolerance was linked to changes in cell composition. Under 2-PE, high-sugar, and high-salt stresses, yeast cells developed stress tolerance mechanisms, primarily involving increased ergosterol content, and adjustments to intracellular fatty acid content and proportions, particularly in the presence of glycerol. Glycerol can be metabolized into dihydroxyacetone phosphate, a precursor of acetyl-CoA, which contributes to ergosterol and fatty acid synthesis (64, 93). Ergosterol serves as a signaling molecule that mitigates ROS effects (17, 94) and strengthens the cell membrane, complementing the role of glycerol in cellular tolerance to 2-PE, high-sugar, and high-salt stresses.

In conclusion, this study demonstrated that a glycerol/H^+^ transporter gene *STL1* mutation highly impacts 2-PE stress tolerance and investigated its underlying mechanism. In addition, the contribution of the Stl1 mutation (F427L) to high salt and sugar stress tolerance was explained. In summary, this study revealed the reasons for the enhanced tolerance to multiple stresses due to the specific mutation of the membrane protein Stl1 (Stl1^F427L^). This provides a theoretical basis for the rational mutation of engineered membrane proteins to improve the robustness of industrial yeast strains. This study will provide new ideas for improving the resistance of *S. cerevisiae* to 2-PE, high salt, high sugar, and cross-complex stresses. Notably, Stl1^F427L^ improved stress tolerance only when the HOG pathway was activated under stress conditions and in the presence of glycerol. Therefore, Stl1^F427L^ might be applied to generate salt-tolerant yeast, sugar-tolerant yeast, or yeast chassis cells designed for organic solvents as productions (e.g., 2-PE and ethanol) or organic solvents as substrates (methanol), highlighting its broad applicability in stress tolerance enhancement.

During the biosynthesis of high value-added “natural” products, yeast encounters stress from products and byproducts such as ethanol produced during anaerobic fermentation. The presence of excess glucose and salt in the environment can also affect the survival of *S. cerevisiae*. In practical production, yeast encounters a combination of these stresses, making it crucial to determine whether Stl1^F427L^ can enhance cross-stress tolerance (such as high sugar, salt, and organic solvent stresses combined). Overall, the present research offers a novel approach to improving yeast strains’ capacity to withstand complex stresses in industrial settings.

## AFTER EPUB

*[This article was published on 16 December 2024 with a labeling error in Fig. 2 and 5. The label was made more specific in the current version, posted on 23 December 2024.]*

## SUPPLEMENTAL MATERIAL

Supplemental material spectrum.00089-24-s0001.docx Figures S1 to S12; Tables S1 to S6.

ASM does not own the copyrights to Supplemental Material that may be linked to, or accessed through, an article. The authors have granted ASM a non-exclusive, world-wide license to publish the Supplemental Material files. Please contact the corresponding author directly for reuse.
