# Supplementary material for: Enhanced multistress tolerance of Saccharomyces cerevisiae with the sugar transporter-like protein Stl1F427L mutation in the presence of glycerol
Source: Microbiol Spectr. 2024 Dec 16;13(2):e00089-24. doi: 10.1128/spectrum.00089-24 (PMC11792538; doi:10.1128/spectrum.00089-24)
Supplement: Supplemental material — Figures S1 to S12; Tables S1 to S6. [file spectrum.00089-24-s0001.docx]

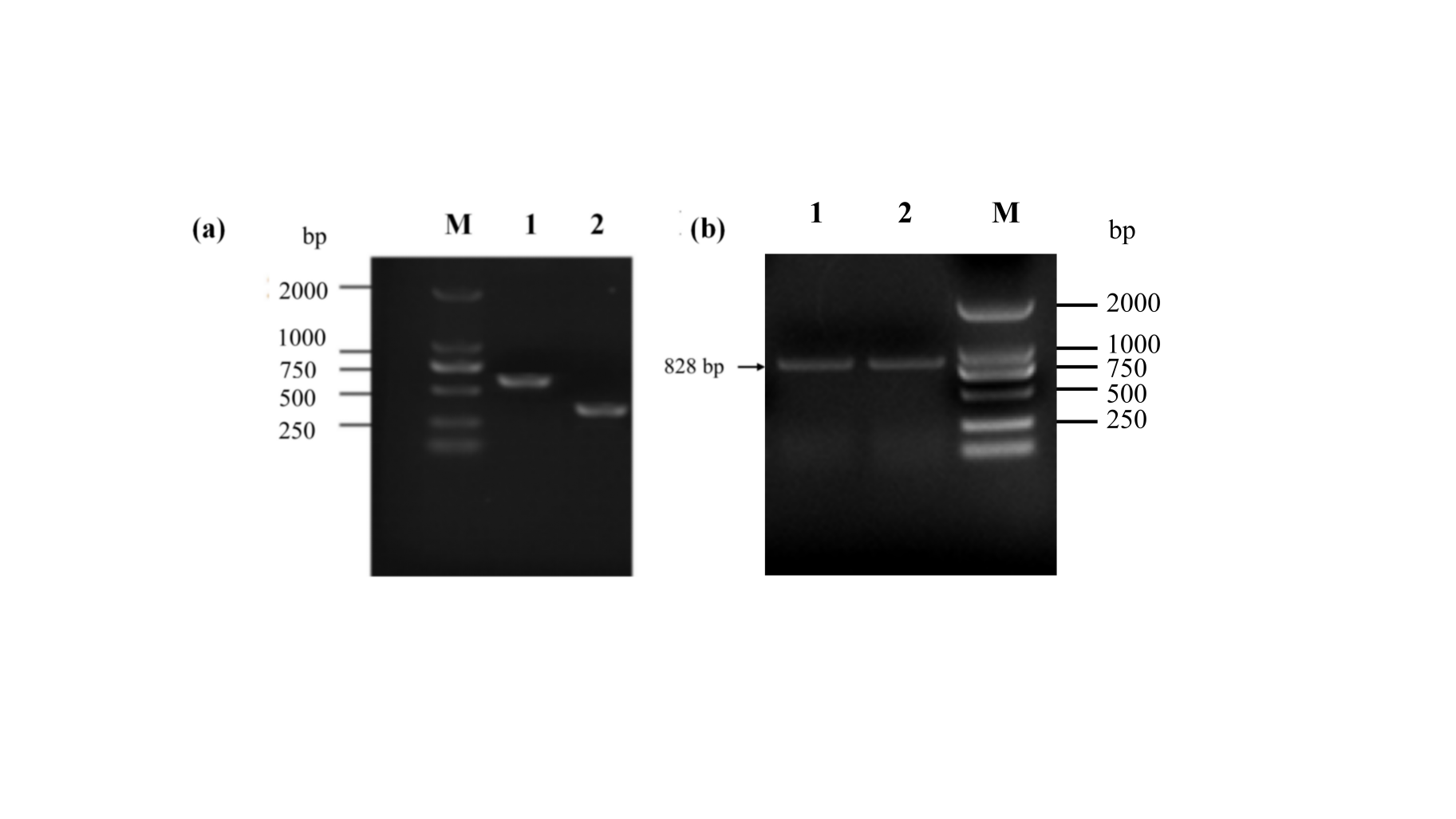


Fig. S1 Construction and PCR validation of *STL1^C1281G^* fragment (Donor DNA).

(a) M: 2000 bp Marker; 1:*STL1^C1281G^* upstream homologous fragment amplification product; 2:*STL1^C1281G^* downstream homologous fragment amplification product

(b) M: 2000 bp Marker; 1/2:*STL1^C1281G^* Donor DNA


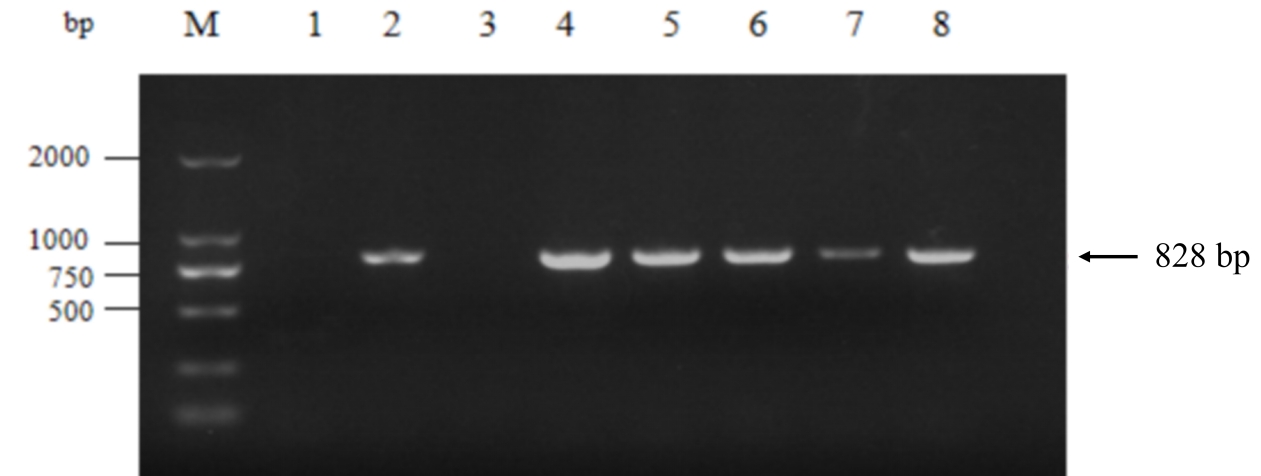


Fig. S2 PCR verification of mutant strains.

M: DNA Marker of DL2000; 2/4/5/6/7/8: PCR amplification of positive clones


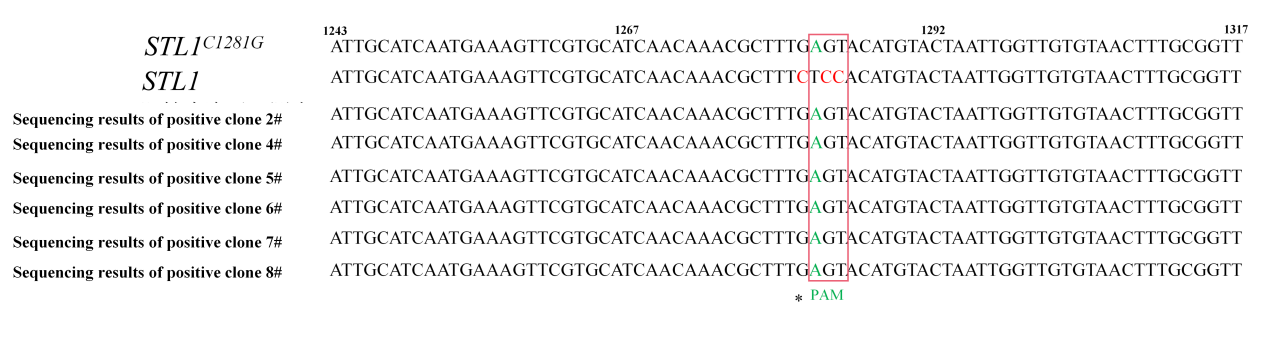


Fig. S3 The mutation of positive clone gene *STL1^C1281G^* was verified by comparison.

* is the mutation site, and PAM is the CRISPR/Cas9 binding site


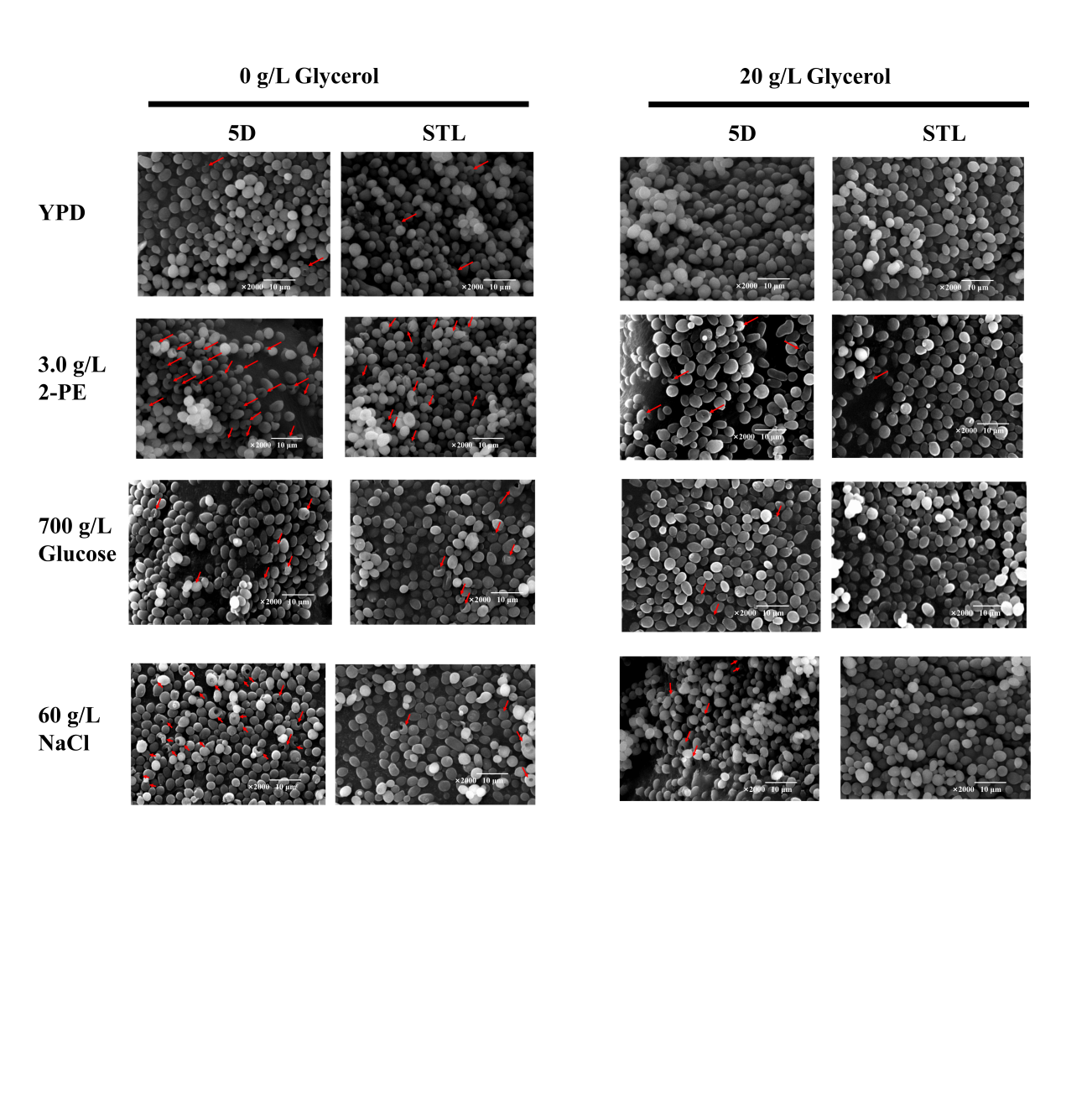


Fig. S4 Comparison of morphological changes of between the control strain 5D and the mutant strain STL. The red arrow pointed to the "doughnut characteristic shape".


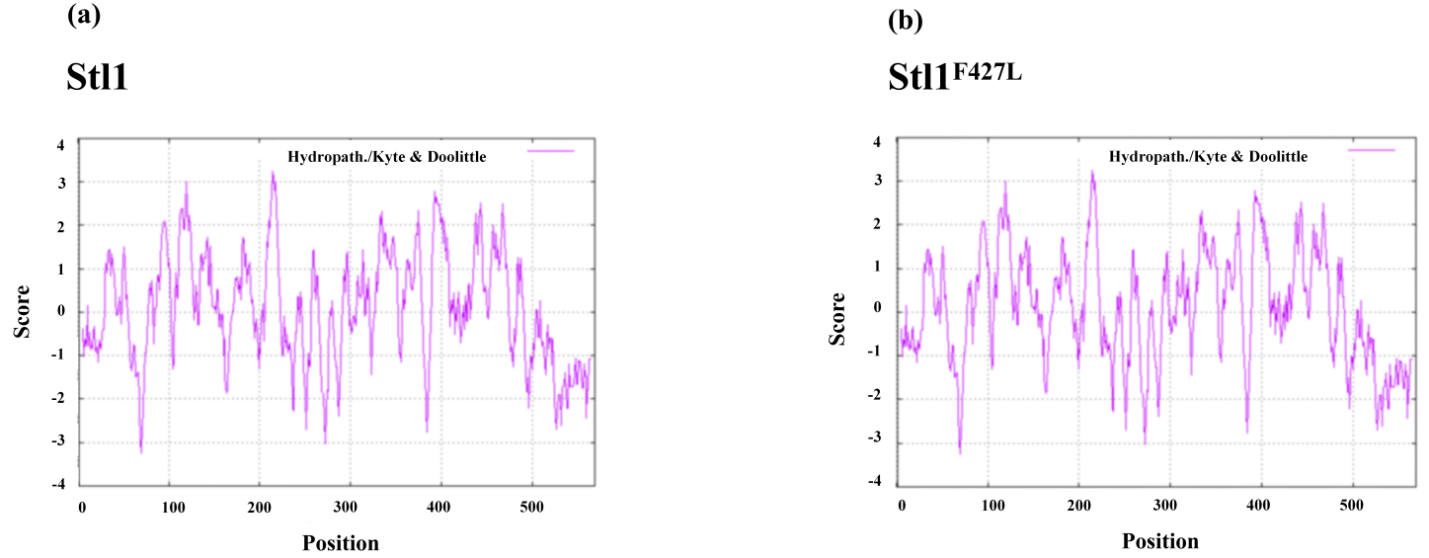


Fig. S5 Prediction of the hydrophobicity/hydrophilicity of Stl1 and Stl1^F427L^ proteins in *S. cerevisiae*.


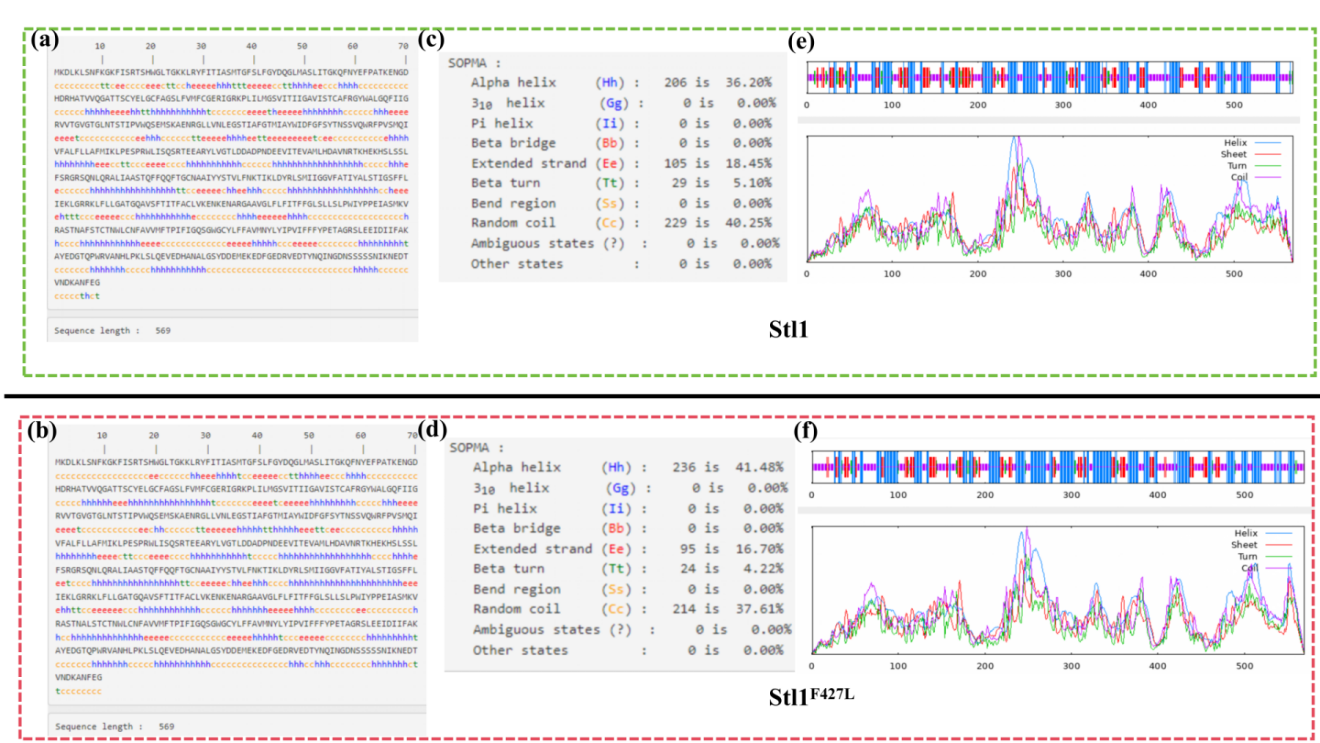


Fig. S6 Prediction of the secondary structure of the Stl1 and Stl1^F427L^ proteins in *S. cerevisiae.*


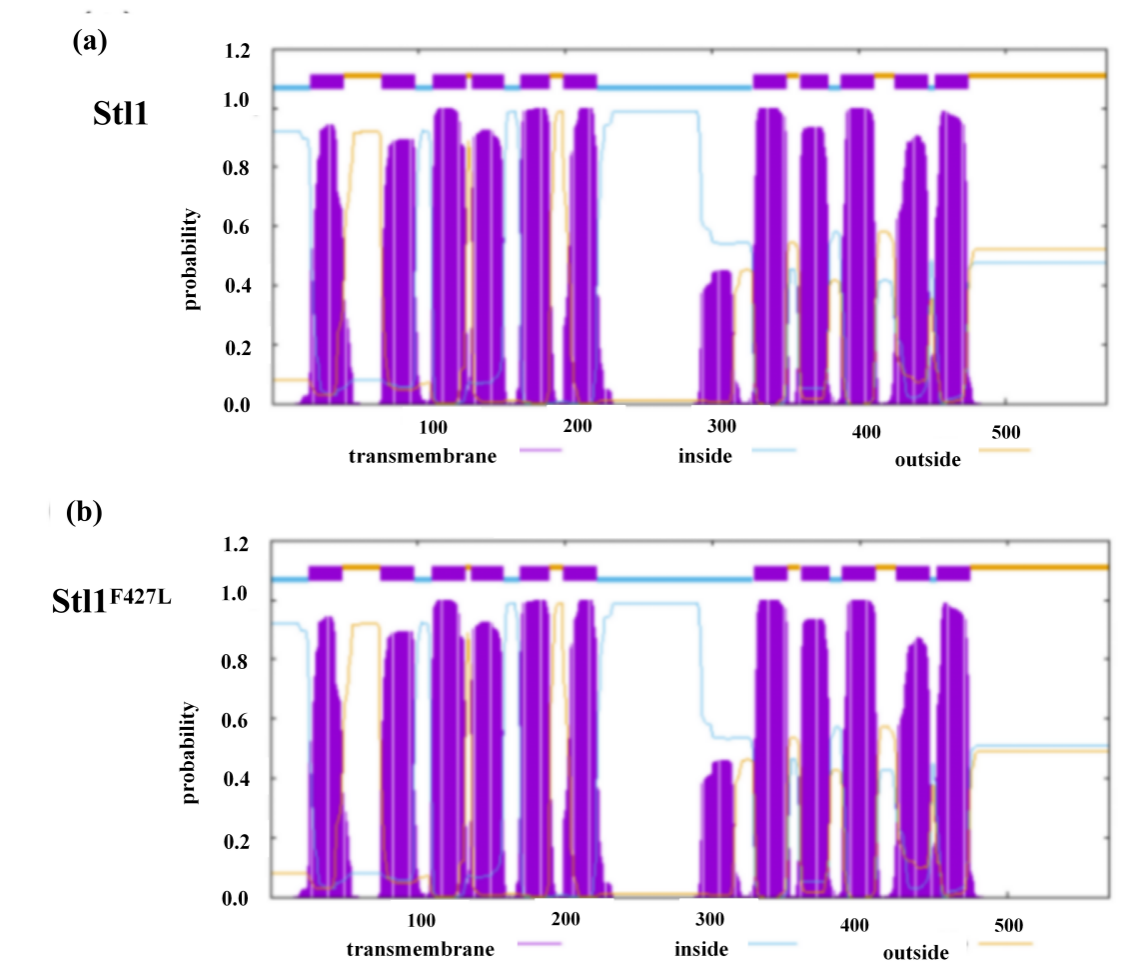
Fig. S7 Prediction of the transmembrane domain of Stl1 and Stl1^F427L^ proteins in *S. cerevisiae.*


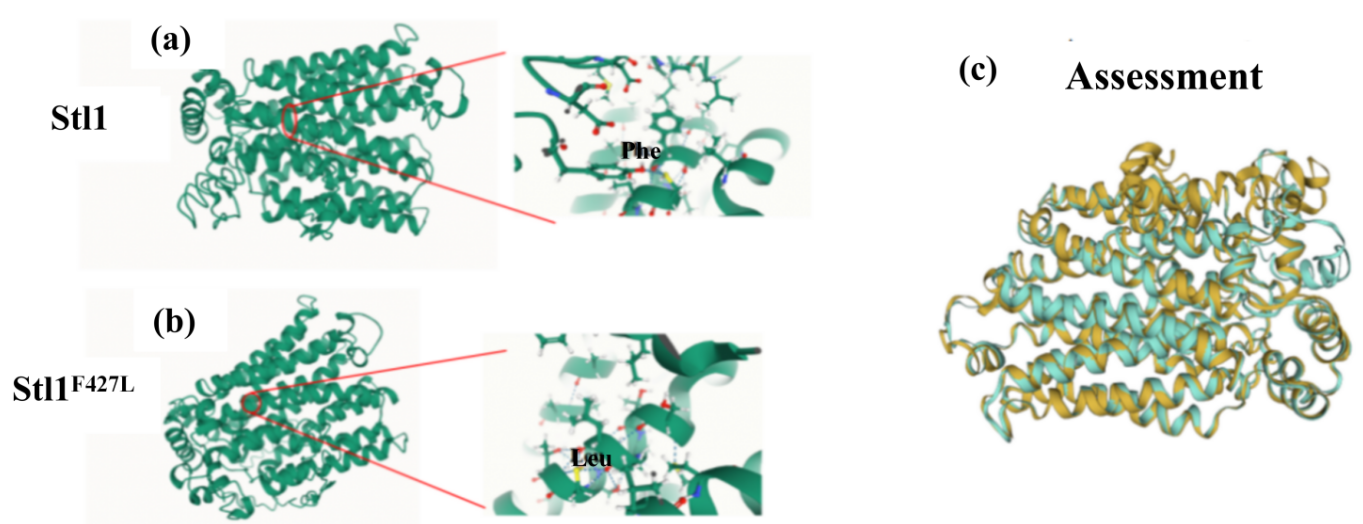


Fig. S8. Prediction and visual comparison of the tertiary structure of Stl1 and Stl1^F427L^ proteins in *Saccharomyces cerevisiae*. (a) depicts the tertiary structure of the Stl1 protein. (b) illustrates the tertiary structure of Stl1 and Stl1^F427L^ proteins. (c) provides a visual comparison of Stl1 and Stl1^F427L^ protein structures. In this panel, the Stl1 protein is represented in blue, and the Stl1^F427L^ protein is represented in yellow.


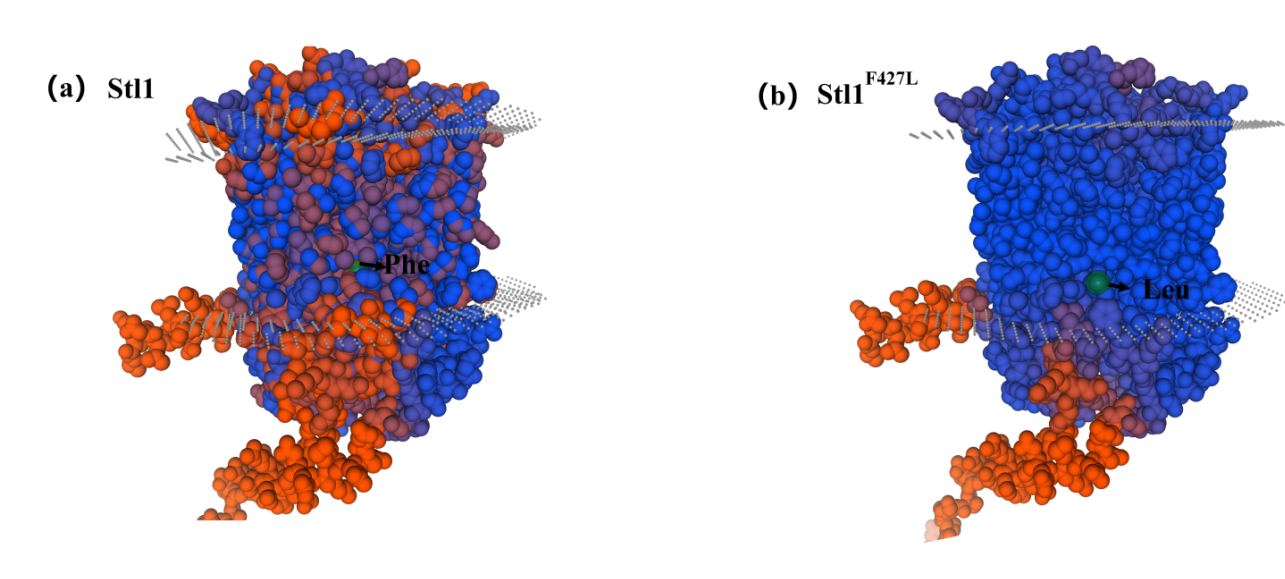


Fig. S9 Spatial filling structure prediction of Stl1 and Stl1^F427L^ proteins in *S. cerevisiae.*

(a) and (b) represent the spatial filling structures of Stl1 and Stl1^F427L^ proteins, respectively


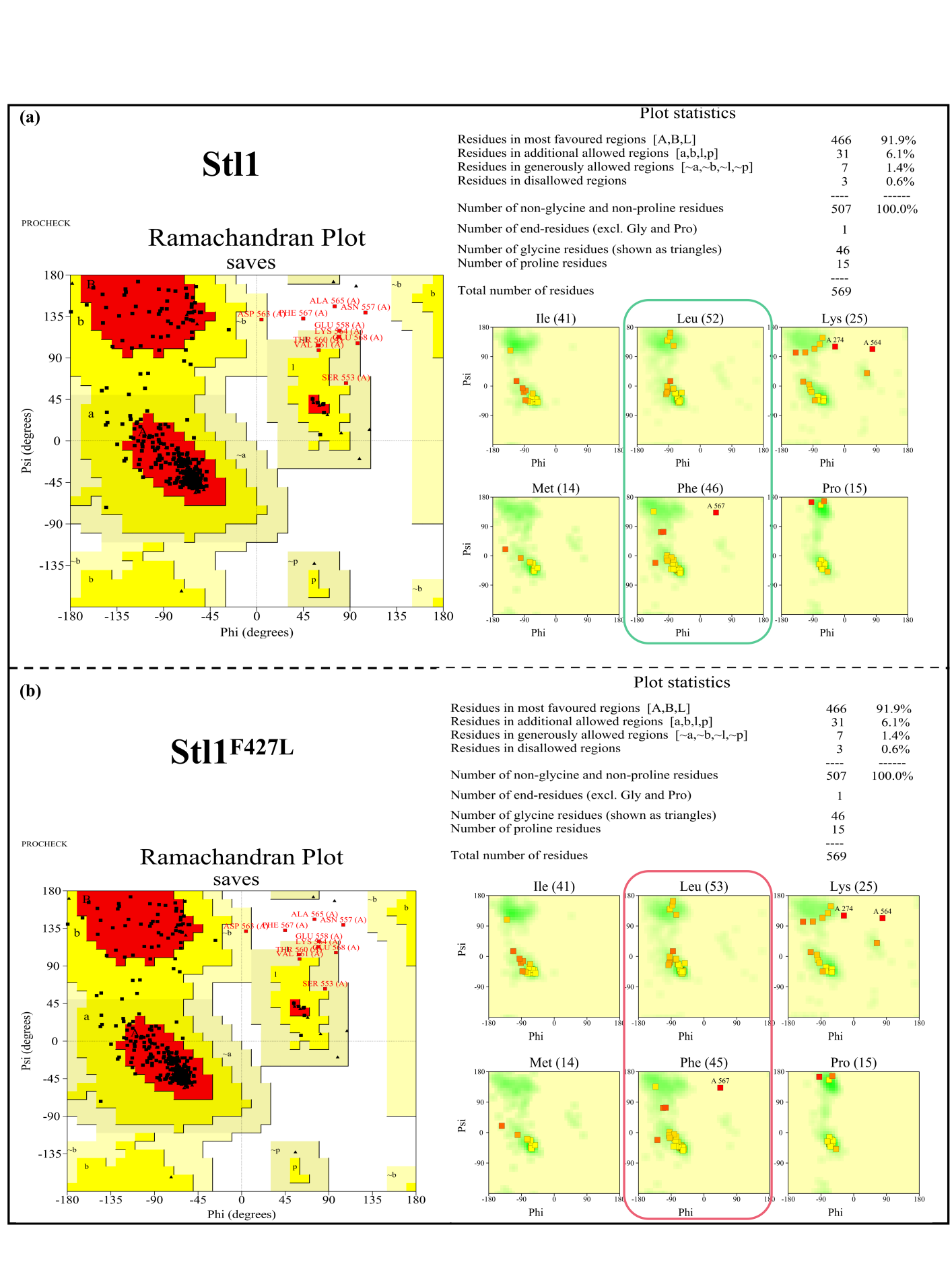


Fig. S10 Ramachandran plot of proteins

(a) Stl1 and (b) Stl1^F427L^


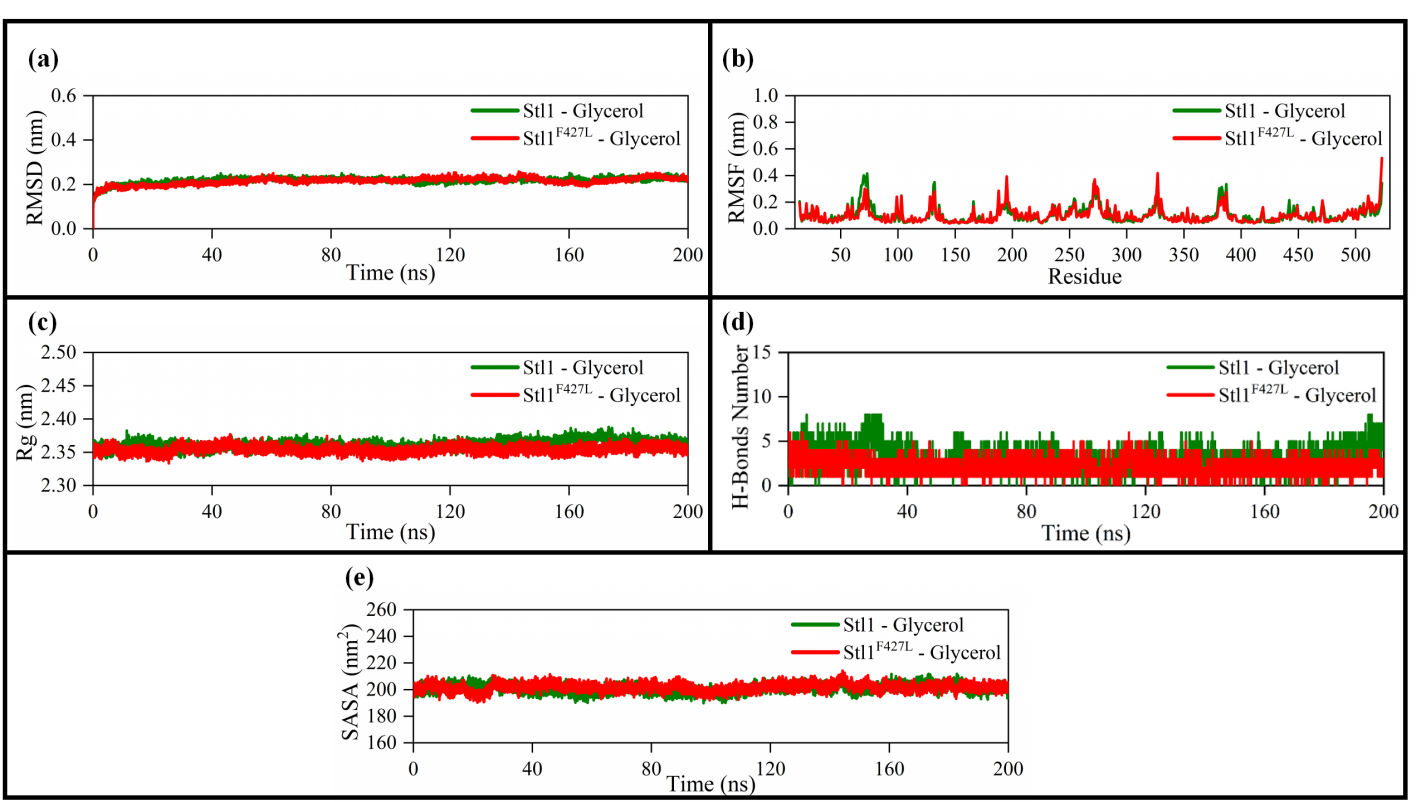


Fig. S11 Molecular dynamics parameters of the complexes formed by Stl1 and Stl1^F427L^ proteins with glycerol, respectively. (a) depicts the changes in root mean square deviation (RMSD) in nanometers (nm) of the complexes. (b) illustrates the changes in root mean square fluctuation (RMSF) in the nm of the complexes. (c) shows the changes in radius of gyration (Rg) in nm of the complexes. (d) displays the changes in the number of hydrogen bonds in the complexes. (e) tells the changes in solvent-accessible surface area (SASA) in nm^2^ of the complexes.


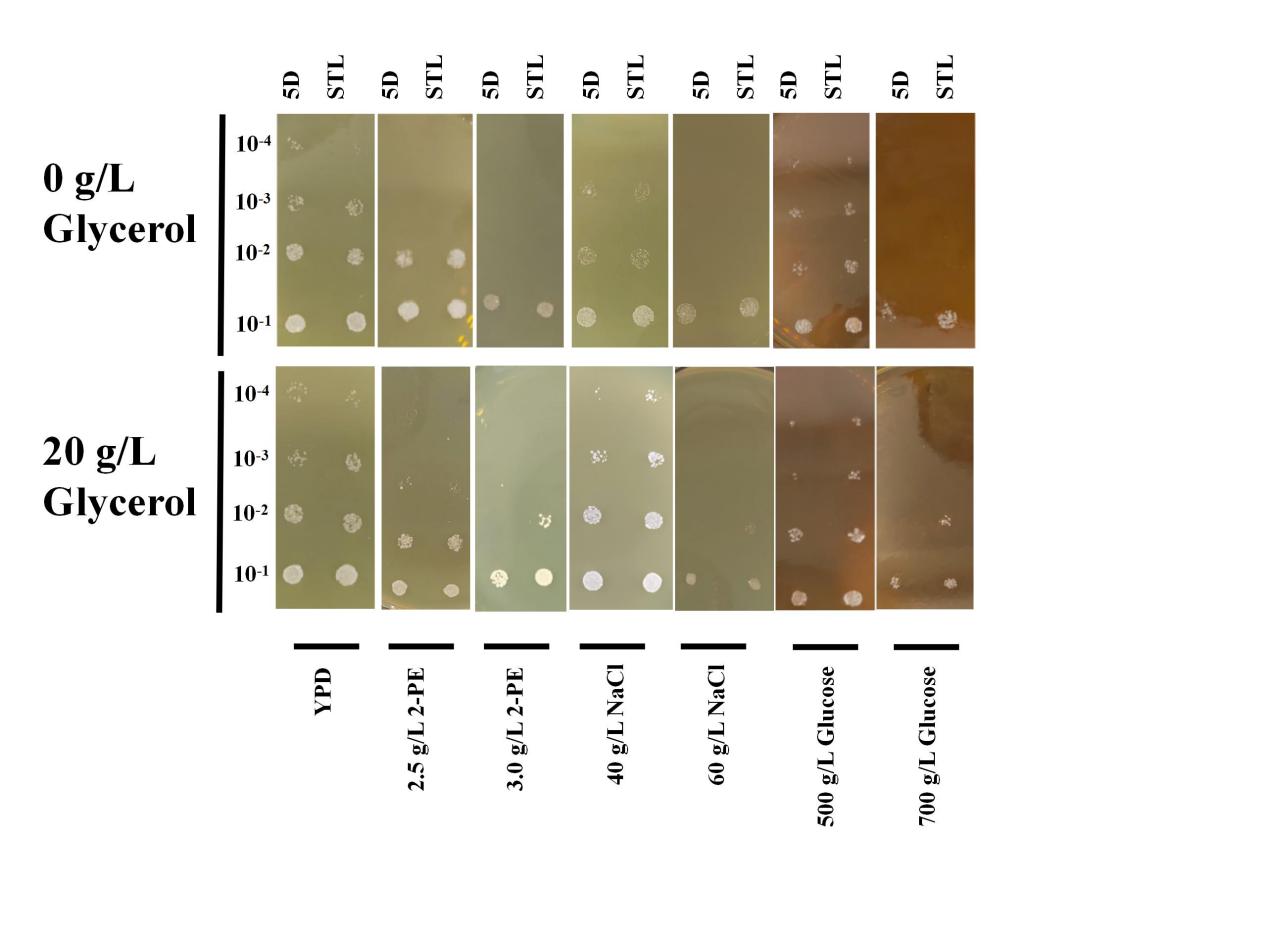


Fig. S12. The original combination figure of Figure 1 in main text.

Table S1. Primer sequences utilized in this experiment.

| Primer name | Primer sequence (5′→3′) | Size (bp) |
| --- | --- | --- |
| *STL1* U-F | GTTGCTATGCTTCACGATGCTGTTAACAGG | 30 |
| *STL1* D-F | AAACGCTTTGAGTACATGTACTAATTGGTTGTGT | 34 |
| *STL1* U-R | ACACAACCAATTAGTACATGTACTCAAAGCGTTT | 34 |
| *STL1* D-R | ATCTTCACCAAAGTCCTCTTTTTCCATTTCGTC | 33 |
| *STL1* gRNA- f | GATCACACAACCAATTAGTACATGGTTTTAGAGCTAG | 37 |
| *STL1* gRNA-r | CTAGCTCTAAAACCATGTACTAATTGGTTGTGT | 33 |
| T_3_ Primer | ATTAACCCTCACTAAAG | 17 |

Table S2. Procedure for the heating of the column temperature box.

|  | Rate (℃/min) | Temperature (℃) | Retention time (min) |
| --- | --- | --- | --- |
| 1 | - | 35.0 | 0.50 |
| 2 | 25.00 | 195.0 | 0.00 |
| 3 | 1.00 | 200.0 | 0.00 |
| 4 | 0.50 | 210.0 | 0.00 |
| 5 | 8.00 | 230.0 | 13.60 |

Table S3 Primer for RT-qPCR

| Gene | Primer names | Sequence (5’–3’) |
| --- | --- | --- |
| *ENO1* | *ENO1*-F | GCTATCAAGGCTGCTGGTCA |
|  | *ENO1*-R | CAGCGAAAACAGCGTTGTCA |
| *STL1/STL1^C1281G^* | *STL1**-F | GAAGAGTCGTCACCGGTGTT |
|  | *STL1**-R | ATGGCAGCGTTACAACCAGT |

Table S4. Comparison of physiological parameters in the control strain 5D and mutant strain STL. * *P* < 0.05; ** *P* < 0.01; **** *P* < 0.0001; ns, not significant (one-way ANOVA with Tukey’s multiple comparison). The specific growth rate is denoted as μ (h^−1^) and the maximum OD_600 nm_ is denoted as OD_600 nm_max.

| Glycerol(g/L) | Media | Strains | Lag phase (h) | μ (h^−1^) | OD_600 nm_max |
| --- | --- | --- | --- | --- | --- |
| 0 | Ctrl | 5D | 0 | 0.090 ± 0.0008 | 37.2 ± 1.36 |
|  |  | STL | 0 | 0.088 ± 0.0017 | 34.5 ± 2.78 |
|  | 2-PE | 5D | 0 | 0.090 ± 0.0020 | 4.3 ± 0.21 |
|  |  | STL | 0 | 0.093 ± 0.0022 | 4.7 ± 0.25 |
|  | Glc | 5D | 12 | 0.089 ± 0.0007 | 11.7 ± 0.29 |
|  |  | STL | 24 | 0.090 ± 0.0010 | 12.4 ± 0.43 |
|  | NaCl | 5D | 24 | 0.070 ± 0.0031 | 6.7 ± 0.50 |
|  |  | STL | 12 | 0.069 ± 0.0014 | 7.1 ± 0.36 |
| 20 | Ctrl | 5D | 0 | 0.095 ± 0.0013 | 48.2 ± 2.89 |
|  |  | STL | 0 | 0.093 ± 0.0012 | 43.1 ± 2.54 |
|  | 2-PE | 5D | 0 | 0.074 ± 0.0017 | 7.2 ± 0.44 |
|  |  | STL | 0 | 0.079* ± 0.0010 | 8.6* ± 0.31 |
|  | Glc | 5D | 24 | 0.057 ± 0.0004 | 9.1 ± 0.08 |
|  |  | STL | 24 | 0.093**** ± 0.0003 | 11.6**± 0.07 |
|  | NaCl | 5D | 12 | 0.055 ± 0.0013 | 6.4 ± 0.29 |
|  |  | STL | 24 | 0.084**** ± 0.0005 | 7.2* ± 0.27 |

Table S5. Physicochemical properties of Stl1 and Stl1^F427L^ proteins in *Saccharomyces cerevisiae.*

| Names | Molecular | pI | Negatively charged | Positively charged | Instability | Aliphatic | GRAVY |
| --- | --- | --- | --- | --- | --- | --- | --- |
|  | weight |  | residues | residues | index | index |  |
| Stl1 | 63565.86 | 6.13 | 53 | 49 | 29.04 | 87.10 | 0.093 |
| Stl1F427L | 63531.84 | 6.13 | 53 | 49 | 29.04 | 87.79 | 0.095 |

Table S6. Secondary structure of Stl1 and Stl1^F427L^ proteins in *Saccharomyces cerevisiae*.

| Names | α-helix/ | Extended | β-turn/% | Random |
| --- | --- | --- | --- | --- |
|  | % | strand/% |  | coil/% |
| Stl1 | 36.20% | 18.45% | 5.10% | 40.25% |
| Stl1F427L | 41.48% | 16.70% | 4.22% | 37.61% |
